# Comparing health literacy and behavioral changes in adulthood: A pilot study on alumni of a single school cancer education program

**DOI:** 10.1371/journal.pone.0304381

**Published:** 2024-05-29

**Authors:** Masanari Minamitani, Atsuto Katano, Tsukasa Sasaki, Satoshi Yamaguchi, Keiichi Nakagawa

**Affiliations:** 1 Department of Comprehensive radiation oncology, The University of Tokyo, Bunkyo-ku, Tokyo, Japan; 2 Department of Radiology, The University of Tokyo Hospital, Bunkyo-ku, Tokyo, Japan; 3 Laboratory of Health Education, Graduate School of Education, The University of Tokyo, Bunkyo-ku, Tokyo, Japan; 4 Unit for Mental Health Promotion, Research Center for Social Science & Medicine, Tokyo Metropolitan Institute of Medical Science, Setagaya-ku, Tokyo, Japan; University of Minnesota School of Dentistry, UNITED STATES

## Abstract

Cancer education programs are regularly conducted in schools in Japan. Previous reports on their effectiveness were based on surveys conducted several months after the education. We aimed to evaluate whether cancer education for children influenced their behavioral changes in adulthood. We targeted schools where the Japan Cancer Society has conducted the programs since 2011 and the program participants are older than 21 years currently. Invitations were sent to the participants and controls who graduated one year before or after the participants, and answers were obtained using an online questionnaire. Data were collected on the willingness to undergo cancer screening, sociodemographic characteristics, healthy behaviors, and health literacy. We compared the data from the program participants with those from controls. We requested cooperation from 13 schools; however, only one agreed. The common reason for refusal was privacy concerns. In the all-boys participating school in Tokyo, there was no significant difference in background information between the cancer education (38 participants) and control (55 participants) groups. Multiple linear regression showed that healthcare or welfare education background (β = 0.25, p = 0.01) and health literacy (β = 0.24, p = 0.02) were significant predictors of cancer screening intention, while the presence of cancer education (p = 0.25) was not. Despite severe selection bias, this is the first study to examine the long-term impacts of cancer education. We found no significant impacts on the measured outcome. However, the educational content at that time differed from that of today, and the program’s efficacy should not be negated.

## Introduction

The Japanese cancer education program began as part of the national school health curriculum following the revision of the Cancer Control Act in 2016 and a partial change in the Third Basic Plan to Promote Cancer Control Programs in 2018 [1, 2]. The national program was implemented in phases, starting with elementary schools in 2020, followed by junior high schools in 2021, and high schools in 2022. Before this initiative, formal cancer education was not widespread, and it is assumed that a few schools dealt with cancer independently. The two objectives of this program are to properly understand cancer and subjectively appreciate the values of life and health [2]. In Japan, cancer has been the leading cause of death since 1981 and the age-standardized incidence rate is increasing [3].

Health education is defined as consciously constructed learning opportunities involving some form of communication designed to improve health literacy, including knowledge and life skills conducive to individual and community health [4]. Health literacy is an essential component of school education and is becoming increasingly important [5]. As Japanese people are reported to have limited health literacy, we hypothesized that cancer education might improve it [6, 7]. Previous reports showed partial enhancement of cancer knowledge and screening motivation after cancer education programs [2, 8–10]. However, these studies were conducted at a gap of few months after the program and not after a longer interval. Although evidence-based prevention and screening for cancer are established, we need to verify whether cancer education programs impact participants’ behavioral change after they reach adulthood [11, 12].

Prospective evaluation of cancer education programs in the long-term is challenging because the Japanese government has already implemented them nationwide [13]. The Japan Cancer Society has been conducting cancer lecture programs (doctor visits) since 2011 [14]. This program did not have a rigidly-prescribed instructional content. However, as endorsed by the Japan Cancer Society, the core concept aimed to unify the message across junior high schools in Japan, emphasizing critical points such as the lifetime risk of cancer, the importance of a healthy lifestyle and regular cancer screenings, the comparatively low screening uptake rates in Japan versus Europe and the U.S., and the specifics of HPV vaccination and cervical cancer screening starting ages. This concept guided visiting lecturers, including oncologists, nurses, and cancer survivors, to share their clinical experiences and insights n various formats such as lectures, workshops, and group activities, tailoerd to effectively educate children with the target grades. The sessions typically lasted between one and three hours on a single day.

The purpose of this study was to evaluate the long-term impacts of these programs by comparing the cancer screening histories and intentions of people who had attended these lectures with those who did not during their school days.

## Materials and methods

We targeted schools in which the Japan Cancer Society has been organizing cancer education programs since 2011. The inclusion criteria for schools were as follows: 1) the participants enrolled in the programs were older than 21 years old on March 31, 2022; and 2) the schools, including the alum associations, agreed to participate in the survey and cooperated in sending the survey invitations to the eligible graduates. The exclusion criteria were as follows: 1) the schools were not age-uniform because they were part-time schools; 2) only a subset of the students in the target grade (e.g., only female students in a co-educational school) attended the programs; 3) the details of the students were unclear; and 4) the programs continued for more than two years. We analyzed the data for all participants who consented to the survey and answered all required questions.

A questionnaire was developed for the survey by referring to previous studies. We incorporated questions about the willingness to undergo cancer screening and a history of cervical cancer screening (only for women), which is recommended for women aged 20 years and above [12]. Specifically, we added a novel question to gauge participants’ attitudes towards future cancer screenings, utilizing a 6-point Likert scale for detailed insights. This question was formulated as follows:

Please select the attitude, most applicable to you, on undergoing cancer screening in the future.

(1, very low; 2, moderately low; 3, slightly low; 4, slightly high; 5, moderately high; 6, very high)

Other items were sex, age, residence (classified into predominantly urban, intermediate rural, and predominantly rural according to the Organisation for Economic Co-operation and Development regional typology), working status, academic background, medical history, health literacy, and five health-related behaviors (smoking, drinking, diet, physical activity, and body shape) [7, 11, 15, 16]. Considering that educational background, particularly healthcare or welfare, could significantly influence participants’ perspectives on health, we sought detailed responses in this area. We used the Japanese translation of the short version of the European Health Literacy Survey Questionnaire (HLS-Q12), a popular scale for assessing health literacy on a scale of 0–50 [7, 15]. Health-related behaviors were cancer prevention lifestyle recommendations by the Ministry of Health, Labor, and Welfare of Japan [1, 11].

The cooperating schools sent survey invitations to the addresses of eligible graduates, who participated in the cancer education programs (cancer education group) and to those who were in one grade below or above the study participants (control group). After receiving the letter, eligible participants accessed the web page mentioned in the letters and reviewed the explanatory documents. They provided their consents electronically and answered approximately 30 close-ended questions. They received 500 Japanese yen as a reward after completing the survey. The questionnaire and methodology used in this study were approved by the Institutional Review Board of the Graduate School of Medicine and Faculty of Medicine, University of Tokyo (2021330NI).

We compared the data for participants in the cancer education group to those in the control group. We set five items of a healthy lifestyle, which are nationally recommended specifically for cancer prevention, as binary variables of recommended (1) and non-recommended (0): smoking (recommended: never smoked), drinking alcohol (recommended: weekly alcohol consumption of < 150 g), exercising (recommended: ≥ 37.5 and ≥ 31.9 metabolic equivalent hours per day for men and women, respectively), eating salted meals (recommended: consumption of < 0.67 g of fish roe per day), and being overweight (recommended: body mass index [BMI] within the range of 21–27 for men and 19–25 for women); these thresholds were based on a previous Japanese study [11]. The term “recommended” was used only for categorizing responses in our analysis and not included in the actual survey questions. We defined the total score as the healthy behavior score. Pearson’s correlation coefficients between the intention to undergo cancer screening and health literacy score were calculated. To identify practical factors affecting the history and intention to undergo cancer screening, we conducted a t-test and multiple linear regression analysis. We used a half-split approach for categorizing healthy behavior scores, dividing the dataset into two equal halves from the lower values. The independent variables for the multiple linear regression analysis were history of cancer education, health literacy score, and variables that were significant in the t-test. All analyses were performed using the SPSS ver. 27, and the significance level was set at 5%.

## Results

The survey was conducted between April 1 and December 16, 2022. We sent requests for cooperation to 13 schools that met the inclusion criteria on April 1, 2022. Only one all-boys school agreed to participate, and we commenced sending survey invitations to eligible participants from this school on April 28, with a deadline of May 31. Despite our continued efforts, the remaining 12 schools declined to participate by December 16, 2022, at which point our data collection process closed. Table 1 describes the reasons for refusing to participate. The main reasons were privacy concerns (33%), staff shortages (25%), and no specific reasons (42%).

**Table 1:** School cooperation details.

School characteristics				Cancer education details				Survey details	
ID	Type	Sex	Location	Year	Target grade	Number of participants	Lecturer	Survey cooperation	Reasons for refusal
1	Private	M	Ur (Tokyo)	2013	JH2	200	Dr and Su	Yes	
2	Private	W	Ur (Kanagawa)	2011	JH3, SH1	430	Dr	No	Privacy policy
3	Public	M/W	Ur (Kanagawa)	2012	SH1	280	Dr	No	No specific reason
4	Private	M/W	IR (Okayama)	2012	SH1, SH2, SH3	100	Dr	No	No specific reason
5	Private	W	Ur (Hyogo)	2012	SH1, SH2, SH3	1300	Dr and Su	No	Privacy policy
6	Private	W	Ru (Nagano)	2013	SH3	179	Dr	No	Privacy policy
7	Public	M/W	Ur (Hyogo)	2013	JH3	216	Dr	No	No specific reason
8	Public	M/W	IR (Kagoshima)	2013	SH1, SH2, SH3	216	Dr	No	Staff shortages
9	Public	M/W	Ru (Shimane)	2014	JH2	97	Dr	No	Staff shortages
10	Public	W	IR (Gunma)	2014	SH1	325	Dr	No	No specific reason
11	Public	M/W	Ur (Chiba)	2015	SH5	143	Dr	No	No specific reason
12	Public	M/W	Ur (Kanagawa)	2015	JH3	140	Dr	No	Privacy policy
13	Public	M/W	Ru (Iwate)	2015	SH1, SH2, SH3	106	Dr	No	Staff shortages

Abbreviations: M: Men, W: Women, Ur: Predominantly Urban, IR: Intermediate Rural, Ru: Predominantly Rural

JH: Junior-High, SH: Senior-High, Dr: Docter, Su: Cancer Survivor

We sent survey letters to 600 graduates of this cooperative school, and 93 responded by the end of the survey one month later. Thirty-eight participants (41%) were classified into the cancer education group and 55 (59%) into the control group.

Table 2 presents the background and intention to undergo cancer screening. Screening for cervical cancer could not be measured because all respondents were men. There were no marked differences in demographic background, health behaviors, or HLS-Q12 scores between the two groups. The typical medical histories were bronchial asthma (10%) and mental health problems (3%); however, none had a history of cancer. Among the respondents, 95% in the cancer education group and 89% in the control group intended to screen for cancer.

**Table 2:** Participants’ characteristics and cancer screening intention.

		Cancer education group		Control group	
		(Age: 22–23, N = 38)		(Age: 21–24, N = 55)	
		N	%	N	%
Sex					
	Men	38	100%	55	100%
Living location					
	Tokyo	30	79%	41	75%
	Outside Tokyo	8	21%	14	25%
Living situation					
	Living alone	6	16%	12	22%
	Living with someone	32	84%	43	78%
Occupational status					
	Student	31	82%	48	87%
	Employed	7	18%	7	13%
Last or current educational background					
	Healthcare or welfare	10	26%	22	40%
	Not healthcare or welfare	28	74%	33	60%
Past medical history					
	Present	10	26%	11	20%
	Absent	28	74%	44	80%
Family cancer history					
	Zero	27	71%	38	69%
	Any	11	29%	17	31%
Smoking					
	Recommended	37	97%	49	89%
	Non-recommended	1	3%	6	11%
Drinking alcohol					
	Recommended	34	89%	51	93%
	Non-recommended	4	11%	4	7%
Exercising					
	Recommended	5	13%	13	24%
	Non-recommended	33	87%	42	76%
Eating salted meals					
	Recommended	35	92%	50	91%
	Non-recommended	3	8%	5	9%
Being overweight					
	Recommended	22	58%	31	56%
	Non-recommended	16	42%	24	44%
Healthy behavior score					
	4–5	21	55%	27	49%
	0–3	17	45%	28	51%
HLS-Q12 score					
	Mean, (SD)	26.1	(7.11)	26.6	(8.77)
Intention to undergo cancer screening					
	Very high/ moderately high/ slightly high	36	95%	49	89%
	Very low/ moderately low/ slightly low	2	5%	6	11%

Abbreviations: HLS-Q12: the short version of the European Health Literacy Survey Questionnaire

Recommended cut-off points: never smoked (smoking), weekly alcohol consumption of < 150 g (drinking alcohol), ≥ 37.5 and ≥ 31.9 metabolic equivalent hours per day for men and women, respectively (exercising), consumption of < 0.67 g of fish roe per day (eating salted meals), body mass index within the range of 21–27 for men and 19–25 for women (being overweight).

The Pearson’s correlation coefficient between cancer screening intention and health literacy score was 0.252, showing a weak correlation. Table 3 presents a significant difference in the screening intention based on educational background (healthcare or welfare: 5.22, not healthcare or welfare: 4.72, p = 0.02). However, there was no difference based on other parameters, as well as on the presence of cancer education (cancer education group: 4.97, control group: 4.84, p = 0.50).

**Table 3:** T-test results: Comparison of cancer screening intention based on each variable.

		Intention to undergo cancer screening	p value
Group			0.50
	Cancer education group	4.97	
	Control group	4.84	
Living location			0.68
	Tokyo	4.92	
	Outside Tokyo	4.82	
Living situation			0.80
	Living alone	4.94	
	Living with someone	4.88	
Occupational status			0.65
	Student	4.91	
	Employed	4.79	
Last or current educational background			0.016
	Healthcare or welfare	5.22	
	Not healthcare or welfare	4.72	
Past medical history			0.10
	Present	5.19	
	Absent	4.81	
Family cancer history			0.64
	Zero	4.92	
	Any	4.82	
Healthy behavior score			0.97
	4–5	4.9	
	0–3	4.89	

Table 4 depicts the results of the multiple linear regression analysis, in which the independent variables were cancer education, HLS-Q12 score, and educational background. Multicollinearity was not observed. There were no significant differences in the intention to screen between the two groups (p = 0.25); however, healthcare or welfare education (β = 0.25, p = 0.01) and higher health literacy scores (β = 0.24, p = 0.02) were significantly different.

**Table 4:** Multiple linear regression analysis results: Association of cancer screening intention with other characteristics.

		B value	S.E.	β value	p value
Variable					
	Group (cancer education vs. control)	0.22	0.19	0.12	0.25
	Last or current educational background (healthcare or welfare vs. not healthcare or welfare)	0.50	0.20	0.25	0.01
	HLS-Q12 score	0.03	0.01	0.24	0.02

Abbreviations: S.E.: Standard Error, HLS-Q12: a short version of the European Health Literacy Survey Questionnaire

R^2^ = 0.13, adjusted R^2^ = 0.10 (N = 93, P value < 0.001)

## Discussion

This study evaluated the long-term impacts of cancer education programs by evaluating whether the program in school influences the intention of cancer screening when students reach adulthood. Among the 13 schools that met the inclusion criteria, only one cooperated with the survey and the most common reason for refusal was privacy concerns. The school was for private urban boys; therefore, cervical cancer screening rates at the age of 20 could not be compared. At this school, a cancer education session was conducted on October 22, 2013 [17]. The program was delivered by a radiation oncologist and a cancer survivor. They interacted with 173 second-year junior high school students (ages 13–14). The session consisted of a lecture and a workshop, which used clear and simple language to ensure that the students understand the material. Under the concept of the “doctor visit”, which was described in the introduction, the radiation oncologist focused on cancer progression, prevention, screenings, and treatment options. The survivor shared personal experiences with stomach and thyroid cancer, as well as her son’s experience with leukemia. The workshop segment encouraged students to reflect on the emotional impact of losing loved ones to cancer and to consider preventive measures for themselves and others. A pre-and post-course survey revealed significant improvements in students’ understanding of cancer-related issues: lifetime cancer morbidity (correct response rate, pre 31.0%, post 100%), most common causes of cancer (pre 60.1%, post 97.0%), and cancer preventability (pre 62.5%, post 93.3%) [17]. Attending cancer education programs did not affect cancer screening intention but significantly improved health literacy scores, whereas healthcare or welfare educational background and health literacy scores significantly improved cancer screening intention. A recent Japanese study showed improvement in knowledge of cancer through pre- and post-class evaluations [2]. Other studies have conducted assessments immediately after the cancer education class in the U.S., three months later in Korea, and six months later in the U.K., using original survey sheets, and confirmed improvements in knowledge and psychological aspects [8–10]. However, a study from Korea, which aligns more closely with the focus of our study, assessed healthy behavioral intentions to prevent cancer after an education program in elementary school students and reported no significant improvement after three months [9]. The present study was unique as it involved a longer gap than the previous studies, a gap of approximately 10 years once participants reached adulthood. Most previous efficacy analyses of school health education have focused on smoking, suggesting relatively long-term efficacy; however, no firm conclusions have been established [18]. In this study, we did not assess improvements in cancer knowledge, but cancer screening as a behavioral change. The results showed no significant differences between the cancer education group and the control group.

There are several possible reasons for the negative results of this study. First, in this eligible school, a radiation oncologist conducted cancer education lessons about essential information, prevention, and screening for cancer nine years prior [17]. Japanese people are less likely than people from other countries to seek cancer screening [19]. Although the purpose of cancer education is not only to improve cancer screening rates, this study focused on cancer screening as a surrogate indicator of behavioral change. Cervical cancer screening is recommended for Japanese women from the age of 20 years, and we planned to compare the actual screening rates [12]. However, we were unable to survey women because many schools refused to participate. Our study was restricted to measuring the intention to undergo screening. Observing the actual screening experience, instead of the intention, might have influenced the findings. Second, in this study, the intention to undergo cancer screening in both groups was higher than expected. A previous Japanese survey, which only investigated cancer-screening intention, sources of cancer information, and level of cancer understanding among Japanese adolescents and was not linked to a cancer education program, reported that 68% of students indicated that they were willing to undergo cancer screening, in contrast to 91% in our study, although there were differences in the target age and questionnaires [20]. Moreover, health literacy levels are associated with cancer screening uptake [21]. The HLS-Q12 score for the Japanese is 25–26, while it was 26.4, i.e., slightly higher, in this study [7, 15]. The stronger willingness to screen for cancer may not have caused a significant difference in health literacy. However, the fact that more than one-third of the respondents in this study had healthcare or welfare education correlated with their willingness to screen. Third, in a past review, one-on-one educational approach significantly improved cancer screening rates with more robust evidence than group education because a personalized approach allows for the consideration of each participant’s unique psychological and behavioral characteristics [22]. Cancer education conducted on a group scale may be effective in a small group. However, we should emphasize that although no difference was found based on the one-time cancer education in this study, this does not negate the effect of ongoing cancer education programs currently provided in Japan with more profound and continuous content.

This study has some limitations. First, mainly due to privacy concerns, only one school cooperated, a private all-boys school in Tokyo. Considering the recommendations for routine cancer screenings in Japan, which typically commence at the age of 40, our study’s participants, aged between 21 and 24, may not have perceived the screenings as practical. Approximately 90% of respondents in both the intervention and control groups expressed an intention to undergo cancer screening. However, it is noteworthy that these responses may be influenced by the prevailing healthcare attitudes in Japan, where annual health check-ups are standard [23]. Consequently, participants may have perceived cancer screenings as a routine rather than as a conscious health choice. Furthermore, the survey participation rate of the target schools was approximately 20 percent. This low response rate may have been partly due to the school’s reliance on graduation address lists, which may have had out-of-date or incorrect information. Therefore, some invitations may not have successfully reached the intended participants. Unfortunately, we were not able to determine the exact reasons for the low response rate, as we could not verify which alumni had received the invitations. In addition, respondents’ medical interests may have been generally high. This may have led to a large selection bias. Second, our study faces a potential validity concern regarding participant classification. While we classified respondents into two groups, cancer education and control, by school grade, we could not confirm whether the respondents of the cancer education group participated in the cancer education lecture at that time. According to our records, only 86.5% (173/200) of the students in this group attended the education session. This means that some respondents in the cancer education group may not have participated in the program, which is a limitation that may have affected the interpretability of our findings. Third, the cancer education program was conducted by a radiation oncologist. While adhering partially to the Japan Cancer Society’s concept by covering topics like cancer screening and HPV vaccination, the program allowed for a degree of flexibility in how content was delivered. As a result of the achievements of this program, the current national cancer education programs have became more structured, with clearly defined curriculum guidelines [24]. These programs encompass nine key cancer-related topics: 1) causes of cancer; 2) cancer processes; 3) the current cancer situation in Japan; 4) cancer prevention strategies; 5) cancer screenings; 6) treatment options; 7) palliative care; 8) quality of life for patients with cancer; and 9) understanding and empathizing with patients with cancer [24]. Additionally, these programs are designed to be conducted in every school by school teachers and visiting lecturers, including medical professionals and cancer survivors [24]. The program content at that time was different from what it is today. Fourth, this study was not planned when the "doctor visits" were first introduced. One of the authors, a launch member of this program, had anticipated the potential for future evaluations. Still, a concrete research plan was not established because the primary goals of the Japan Cancer Society were enlightenment and education, without an emphasis on research. However, when formal cancer education programs began across the country around 2020, the importance of the evaluation became more apparent, leading us to conduct this study. We admit that the study was retrospective and contained some arbitrary nature of its methods and interpretation. Nevertheless, this research addresses a critical point of public health education. Again, the participants in the study are extremely limited—single gender, from a single school, with fewer than 100 participants—which restricts the generalizability of the findings. It is also impractical to apply these findings directly to educational curricula. It is important to recognize this study as an initial, pilot effort to provide the basis for future research. While the current research focused on measuring screening uptake and motivation, a pre- and post-intervention survey found improvements in knowledge of cancer morbidity, preventability, and common causes, which was aligned with the objectives of the program [17]. Future studies should consider evaluating both knowledge and behavioral change. Although systemic cancer education has already begun in Japan, continuous evaluation and curriculum modification are essential for enhancement. Given the challenges of retrospective assessment, as represented in this study, a prospective evaluation research would be more desirable. Future studies should consider longitudinal follow-up to assess the sustainability of cancer knowledge gains before and after the programs. In addition, investigating the impact of different types of visiting lecturers on knowledge acquisition could provide valuable insights. Understanding the long-term persistence of knowledge, which is related to behavior, will clarify the factors contributing to the current study’s negative results and enhance the implications drawn from this research. There is an increasing need to evaluate the impacts of cancer education and the relevance of its content within current formal cancer education programs. Despite its limitations, we believe that this study serves as a valuable pioneering effort in this area.

## Conclusions

This study reports a long-term evaluation of the impacts of cancer education programs conducted nine years prior in an all-boys private school. The program was distinctive in its implementation, with a radiation oncologist and a cancer survivor sharing their clinical experiences and insights, respectively, through lectures and workshops. There was no difference in the intention to undergo cancer screening depending on attending the program, while healthcare or welfare educational background and health literacy were significant factors. This study had a few valid participants, mainly due to privacy concerns, which led to a strong selection bias. The content of cancer education programs at that time was not identical to that provided today; therefore, these findings does not necessarily deny the effectiveness of the current programs. We must evaluate these major intervention programs in the school curriculum in the future, after carefully considering the methodology.
