# Supplementary material for: Comparing health literacy and behavioral changes in adulthood: A pilot study on alumni of a single school cancer education program
Source: PLoS One. 2024 May 29;19(5):e0304381. doi: 10.1371/journal.pone.0304381 (PMC11135696; doi:10.1371/journal.pone.0304381)
Supplement: Attachment — Submitted filename: Response to Reviewers.docx [file pone.0304381.s001.docx]

Boyen Huang, DDS, MHA, PhD
Academic Editor
PLOS ONE

Jan 15^th^, 2024

Dear Boyen Huang, and Reveiwers

Subject: Submission of revised paper PONE-D-23-29716 entitled "Evaluating the long-term effectiveness of school cancer education programs on behavior change: a first trial study".

Thank you very much for reviewing our manuscript and offering valuable advice. We have addressed your comments and revised the manuscript accordingly. We modified our manuscript and ordered English-proof reading again.

We would be glad to respond to any further questions and comments that you may have.

Thank you very much for your consideration.

Sincerely,

Masanari Minamitani.

Response to Reviewer #1:

Thank you for your comments. Our answers to your points are as follows.

1) This is important to consider for overall impact of cancer, education, screening and health in general, however, I think there are some limitations to study that may limit the applicability of these results in the real world.

Considering that largely young males were included in the survey portion of the study, there may be significant impact on the results given that this group rarely has needs for screening in general, and I'm unaware of any immediate needs for cancer screening outside of Circle cancer, screening and women. This may influence the results, although it is unclear to me what it is more less likely to respond that they are willing to consider screening in the future.

Answer. Thank you for your insightful comments regarding the limitations of our study, particularly concerning the demographic characteristics of our survey participants and the implications for the real-world applicability of our results.

In our study, we focused on a single private all-boys school in Tokyo for our survey due to challenges in securing cooperation from other schools, primarily by concerns over privacy. We acknowledge that this focus has potentially influenced the outcomes, given that the participant group primarily comprised young males aged 21 to 24 years. This age group is typically less engaged in cancer screening, as the recommendations for routine screenings such as gastric, lung, and colorectal cancer in Japan commence at the age of 40. Consequently, the immediate practicality and relevance of these screenings may not have been apparent to our participants.

Furthermore, it is important to consider the broader context of the Japanese healthcare system, where annual general health checkups are customary. This cultural norm may lead to an inherent expectation of cancer screenings as a routine practice, possibly influencing the high percentage of participants (approximately 90% in both the intervention and control groups) who indicated their intention to undergo cancer screening in the future. However, we recognize that the accuracy of these responses might be subject to bias, as the participants' current age and healthcare norms could diminish the perceived immediacy or personal relevance of such screenings.

To address these points, we have included the following sentences in the limitations section of our manuscript:

A. Considering the recommendations for routine cancer screenings in Japan, which typically commence at the age of 40, our study's participants, aged between 21 and 24, may not have perceived the screenings as practical. Approximately 90% of respondents in both the intervention and control groups expressed an intention to undergo cancer screening. However, it is noteworthy that these responses may be influenced by the prevailing healthcare attitudes in Japan, where annual health check-ups are standard [OECD Reviews of Public Health: Japan A HEALTHIER TOMORROW. OECD Publishing: Paris; 2019.]. Consequently, participants may have perceived cancer screenings as a routine rather than as a conscious health choice. (line 259-266)

2) Relatively high willingness to have any sort of cancer screening in the future, and concerned that this overall willingness to undergo cancer screening also results. it is unclear to me whether this is a Japanese cultural connection, or that overall willingness tend to cancer has risen from what be historical controls.

Answer: Thank you for highlighting the observed high willingness to undergo cancer screening among study participants. In our study, we noted an unexpectedly high inclination among participants in both the intervention and control groups to undergo future cancer screenings. As discussed in our manuscript, we believe this may be attributed to several factors. Firstly, the participants in our study exhibited a slightly higher level of health literacy and more frequently had educational backgrounds in healthcare or welfare compared to the general population. This could inherently influence their attitudes towards health screenings, including cancer screening. Additionally, as mentioned in our previous response (comment 1), the participants, being young males, might perceive cancer screening as a routine aspect of healthcare. To better reflect these considerations, we have revised the discussion section of our manuscript.

A. Second, in this study, the intention to undergo cancer screening in both groups was higher than expected. A previous Japanese survey, which only investigated cancer-screening intention, sources of cancer information, and level of cancer understanding among Japanese adolescents and was not linked to a cancer education program, reported that 68% of students indicated that they were willing to undergo cancer screening, in contrast to 91% in our study, although there were differences in the target age and questionnaires [20]. (line 239-244)

A. Considering the recommendations for routine cancer screenings in Japan, which typically commence at the age of 40, our study's participants, aged between 21 and 24, may not have perceived the screenings as practical. Approximately 90% of respondents in both the intervention and control groups expressed an intention to undergo cancer screening. However, it is noteworthy that these responses may be influenced by the prevailing healthcare attitudes in Japan, where annual health check-ups are standard [OECD Reviews of Public Health: Japan A HEALTHIER TOMORROW. OECD Publishing: Paris; 2019.]. Consequently, participants may have perceived cancer screenings as a routine rather than as a conscious health choice. (line 259-266)

3) It is also unclear to me what the specific intervention was regarding cancer education. The level of detail is sparse, and, as an educationalist, it is unclear to me, whether the lack of impact is due to limitations in the teaching or evaluation methods at the time of teaching. While this is not specifically part of the study, this contextual background is important to understand what Impacts the actual education program itself potentially have on the outcomes of this survey.

Answer: Thank you for pointing out the need for a more detailed description of the specific cancer education intervention implemented in our study. Upon reflection, we recognize the importance of providing a comprehensive context for the educational content and methods employed, as these factors are crucial in understanding the potential impact of the program on the study outcomes.

In response to your feedback, we have thoroughly reviewed the records of the school's cancer education program and have updated the relevant sections of our manuscript to include more specific details about the intervention.

A. This program did not have a rigidly-prescribed instructional content. Instead, visiting lecturers, including oncologists, nurses, and cancer survivors, shared their clinical experiences and insights to the target grades in a way they felt was most appropriate for educating children. The sessions were delivered in various formats, including lectures, workshops, and group activities and typically lasted between one and three hours on a single day. (line 75-80)

A. At this school, a cancer education session was conducted on October 22, 2013 [17]. The program was delivered by a radiation oncologist and a cancer survivor. They interacted with 173 second-year junior high school students (ages 13-14). The session consisted of a lecture and a workshop, which used clear and simple language to ensure that the students understand the material. The radiation oncologist focused on cancer progression, prevention, screenings, and treatment options. The survivor shared personal experiences with stomach and thyroid cancer, as well as her son's experience with leukemia. The workshop segment encouraged students to reflect on the emotional impact of losing loved ones to cancer and to consider preventive measures for themselves and others. A pre-and post-course survey revealed significant improvements in students' understanding of cancer-related issues: lifetime cancer morbidity (correct response rate, pre 31.0%, post 100%), most common causes of cancer (pre 60.1%, post 97.0%), and cancer preventability (pre 62.5%, post 93.3%) [17]. (line 202-212)

Response to Reviewer #2:

Thank you very much. Your comments are appreciated. We modified our manuscript.

Major issues:

1) The paper would be strengthened if the authors provided details on the cancer education programs.

a. What age level (JH-2 is mentioned in the table, but knowing the age level of the students would be beneficial.
b. Was the program a one day, one module, one year event?
c. The authors state the program was delivered by a radiation oncologist. Is it known if the physician communicated in an age-appropriate language to increase the understanding/learning of the students.

Answer: Thank you for pointing out the need for a more detailed description of the specific cancer education intervention implemented in our study. Upon reflection, we recognize the importance of providing a comprehensive context for the educational content and methods employed, as these factors are crucial in understanding the potential impact of the program on the study outcomes.

In response to your feedback, we have thoroughly reviewed the records of the school's cancer education program and have updated the relevant sections of our manuscript to include more specific details about the intervention.

A. This program did not have a rigidly-prescribed instructional content. Instead, visiting lecturers, including oncologists, nurses, and cancer survivors, shared their clinical experiences and insights to the target grades in a way they felt was most appropriate for educating children. The sessions were delivered in various formats, including lectures, workshops, and group activities and typically lasted between one and three hours on a single day. (line 75-80)

A. At this school, a cancer education session was conducted on October 22, 2013 [17]. The program was delivered by a radiation oncologist and a cancer survivor. They interacted with 173 second-year junior high school students (ages 13-14). The session consisted of a lecture and a workshop, which used clear and simple language to ensure that the students understand the material. The radiation oncologist focused on cancer progression, prevention, screenings, and treatment options. The survivor shared personal experiences with stomach and thyroid cancer, as well as her son's experience with leukemia. The workshop segment encouraged students to reflect on the emotional impact of losing loved ones to cancer and to consider preventive measures for themselves and others. A pre-and post-course survey revealed significant improvements in students' understanding of cancer-related issues: lifetime cancer morbidity (correct response rate, pre 31.0%, post 100%), most common causes of cancer (pre 60.1%, post 97.0%), and cancer preventability (pre 62.5%, post 93.3%) [17]. (line 202-212)

2) 2. In the abstract (line 31), it is mentioned that the Japan Cancer Society has conducted these education programs since 2011; however in the introduction (lines 51-53), it is mentioned that the education programs became part of the curriculum in 2016. Before 2016 (which is when the study/control participants were in school), it would be helpful to know if the delivery of information was standardized or more invited guest lecture and variable. For instance, was focus on preventive measure compliance a learning objective/goal of the program; this is not mentioned in the objectives mentioned (lines 54-55).

Answer: Thank you for bringing to our attention the discrepancy in the timeline of cancer education programs as mentioned in introduction. We recognize the need to clarify the distinction between the national cancer education programs and the specific initiatives undertaken by the Japan Cancer Society in our study. In our revised introduction, we have included a more detailed explanation to distinguish these two programs.

A. The Japanese cancer education program began as part of the national school health curriculum following the revision of the Cancer Control Act in 2016 and a partial change in the Third Basic Plan to Promote Cancer Control Programs in 2018 [1,2]. The national program was implemented in phases, starting with elementary schools in 2020, followed by junior high schools in 2021, and high schools in 2022. Before this initiative, formal cancer education was not part of the standard school education in Japan. (line 51-56)

A. This program did not have a rigidly-prescribed instructional content. Instead, visiting lecturers, including oncologists, nurses, and cancer survivors, shared their clinical experiences and insights to the target grades in a way they felt was most appropriate for educating children. The sessions were delivered in various formats, including lectures, workshops, and group activities and typically lasted between one and three hours on a single day. (line 75-80)

3) It would be helpful if the authors were able to comment on if the questionnaire used was mapped to the curricular objectives of the program in which the students participated or if it was based only on general health and cancer related questions (lines 87-88). For instance, were the items included on lines 91-95 included in the curricular program for the students, or are they general health questions.

Answer: Thank you for your inquiry regarding the alignment of our survey questionnaire with the curricular objectives of the cancer education program. Please note that the questionnaire was primarily designed to gather general health and cancer-related information, rather than directly linking to the specific curriculum of the program.

A. A questionnaire was developed for the survey by referring to previous studies. We incorporated questions about the willingness to undergo cancer screening and a history of cervical cancer screening (only for women), which is recommended for women aged 20 years and above [12]. Specifically, we added a novel question to gauge participants' attitudes towards future cancer screenings, utilizing a 6-point Likert scale for detailed insights. This question was formulated as follows:

Please select the attitude, most applicable to you, on undergoing cancer screening in the future.

(1, very low; 2, moderately low; 3, slightly low; 4, slightly high; 5, moderately high; 6, very high)

Other items were sex, age, residence (classified into predominantly urban, intermediate rural, and predominantly rural according to the Organisation for Economic Co-operation and Development’s regional typology), working status, academic background, medical history, health literacy, and five health-related behaviors (smoking, drinking, diet, physical activity, and body shape) [7,11,15,16]. Considering that educational background, particularly healthcare or welfare, could significantly influence participants' perspectives on health, we sought detailed responses in this area. We used the Japanese translation of the short version of the European Health Literacy Survey Questionnaire (HLS-Q12), a popular scale for assessing health literacy on a scale of 0-50 [7,15]. Health-related behaviors were cancer prevention lifestyle recommendations by the Ministry of Health, Labor, and Welfare of Japan [1,11]. (line 96-115)

4) The authors describe the control group as students from one year before and one year after the study group. It would be helpful to explain this in more detail. As written, the reader understands that education programs began in 2011 and more formally in 2016; so, if the education program was ongoing at the time of the study participants, how are students one year afterwards a control group. One year before could be students who did not have the program, but if the program was given in 2013 (table 1), would one year before and one year after students both have been exposed to the education program.

Answer: Thank you for pointing out the need for a clearer explanation regarding the selection of our control group. The 'doctor visit' program, conducted by the Japan Cancer Society, was designed as a one-time, one-day educational session in our study. This format is crucial to understanding the selection criteria for our control group. We excluded schools where the 'doctor visit' was conducted for more than two years, as per our exclusion criteria. This ensured that the program's impact could be isolated to a specific year group within each school. To clarify, the students from one year before and one year after the study participants were chosen as the control group based on the premise that they did not participate in the "doctor visit." As noted in comment (2), we modified the introduction section to explain the details of the “doctor visit.”

5) It would be beneficial to acknowledge that data requirements for t-test and logistic regression were reviewed and met.

Answer: Thank you for your suggestion to explicitly confirm that our data meets the requirements for both t-test and logistic regression analysis in our study. Although we primarily presented the t-test results for simplicity, we acknowledge the importance of demonstrating the adequacy of our data for subsequent multiple regression analysis. In response to your feedback, we have added an additional table in the results section that details the outcomes of the t-test.

| **Table 3. T-test results: comparison of cancer screening intention based on each variable** | | | |  |
| --- | --- | --- | --- | --- |
|  |  | Intention to undergo cancer screening | p value | |
| Group |  |  | | 0.50 |
|  | Cancer education group | 4.97 | |  |
|  | Control group | 4.84 | |  |
| Living location | |  | | 0.68 |
|  | Tokyo | 4.92 | |  |
|  | Outside Tokyo | 4.82 | |  |
| Living situation | |  | | 0.80 |
|  | Living alone | 4.94 | |  |
|  | Living with someone | 4.88 | |  |
| Occupational status | |  | | 0.65 |
|  | Student | 4.91 | |  |
|  | Employed | 4.79 | |  |
| Last or current educational background | |  | | 0.016 |
|  | Healthcare or welfare | 5.22 | |  |
|  | Not healthcare or welfare | 4.72 | |  |
| Past medical history | |  | | 0.10 |
|  | Present | 5.19 | |  |
|  | Absent | 4.81 | |  |
| Family cancer history | |  | | 0.64 |
|  | Zero | 4.92 | |  |
|  | Any | 4.82 | |  |
| Healthy behavior score | |  | | 0.97 |
|  | 4-5 | 4.9 | |  |
|  | 0-3 | 4.89 | |  |

**Table 4. Multiple linear regression analysis results: association of cancer screening intention with other characteristics**

|  |  | B value | S.E. | β value | p value |
| --- | --- | --- | --- | --- | --- |
| Variable |  |  |  |  |  |
|  | Group (cancer education vs. control) | 0.22 | 0.19 | 0.12 | 0.25 |
|  | Last or current educational background (healthcare or welfare vs. not healthcare or welfare) | 0.50 | 0.20 | 0.25 | 0.01 |
|  | HLS-Q12 score | 0.03 | 0.01 | 0.24 | 0.02 |

Abbreviations: S.E.: Standard Error, HLS-Q12: a short version of the European Health Literacy Survey Questionnaire

R^2^ = 0.13, adjusted R^2^ = 0.10 (N = 93, P value < 0.001)

6) On lines 129-130, it would be helpful to further explain. Were all surveys completed by May 1, or was the letter an invitation to participate and survey sent later. This will help the reader better understand the timeline set in line 125.

Answer: Thank you for your attention to the details of our survey timeline. We recognize the importance of clearly outlining the sequence of events for a better understanding of our study's methodology. We started to ask 13 candidate schools to cooperate our study on April 1^st^, 2022. One school agreed to cooperate, and we began sending invitations to eligible participants on April 28, with a deadline of May 31. The final contact for refusal by school was made on December 16, and the investigation was terminated at that point. We have included the following revised sentences in the results section.

A. The survey was conducted between April 1 and December 16, 2022. We sent requests for cooperation to 13 schools that met the inclusion criteria on April 1, 2022. Only one all-boys school agreed to participate, and we commenced sending survey invitations to eligible participants from this school on April 28, with a deadline of May 31. Despite our continued efforts, the remaining 12 schools declined to participate by December 16, 2022, at which point our data collection process closed. Table 1 describes the reasons for refusing to participate. (line 146-152)

7) It would be helpful if the authors provide more detail on what “medical and welfare” educational background is, as the reader is not familiar with these terms.

8) On table 2, what is “past medical history” with options of “zero” or “any”?

Answer: Thank you for the suggestion regarding the clarity of the word. I have followed your advice and replaced the words "medical and welfare" with "healthcare or welfare " and "not healthcare or welfare.” Also, I have changed the options for "past medical history" to "present" and "absent".

9) Lines 216-217, as written, the reader understands this to mean that the validity of the participants in the study/control groups could be compromised. Therefore, it would be extremely helpful to reword and further explain the meaning.

Answer: Thank you for raising the need for a clearer explanation of the validity of the participants in our study. In our manuscript, we initially classified the respondents into two groups: those in the grade that received the cancer education program (cancer education group) and control group. However, upon further consideration, we recognize a potential limitation in this classification. Not all students classified in the cancer education group may have been present at the cancer education lecture. Records of attendance show that only 173 out of 200 students in the relevant class took part in the program, which means that approximately 13.5% of these students missed it. The limitations section of our manuscript has been revised to address this concern.

A. In addition, respondents' medical interests may have been generally high. This may have led to a large selection bias. Moreover, our study faces a potential validity concern regarding participant classification. While we classified respondents into two groups, cancer education and control, by school grade, we could not confirm whether the respondents of the cancer education group participated in the cancer education lecture at that time. According to our records, only 86.5% (173/200) of the students in this group attended the education session. This means that some respondents in the cancer education group may not have participated in the program, which is a limitation that may have affected the interpretability of our findings. (line 271-279)

10) Lines 226-227, as written the sentence is very lofty as it refers to “cancer education programs” in general, and this study is limited to one school, one program (which may not be similar to current programs?). It would be helpful to reword and focus on the implications of this studying while also understanding the limitations.

Answer: Thank you for pointing out the potential overgeneralization in our conclusions. We agree that it is crucial to accurately represent the scope of our study and its implications, particularly considering that our evaluation was based on a program conducted in a single all-boys private school. We have revised our conclusion.

A. This study reports a long-term evaluation of the effectiveness of cancer education programs conducted nine years ago in an all-boys private school. The program was distinctive in its implementation, with a radiation oncologist and a cancer survivor sharing their clinical experiences and insights, respectively, through lectures and workshops. (line 294-297)

Minor issues
1) The authors may want to revisit the phrasing of the sentence on lines 56-57. The incidence of cancer could be due to genetic factors or factors outside the influence of the education programs. However, the mortality rate could be influenced by preventive screening.

Answer: We revised the sentence as follows to reflect these aspects more accurately.

A. Cancer education programs are expected to promote lifestyle change that reduce cancer incidence and to enhance cancer screening uptake, leading to a decrease in mortality rate. (line 59-61)

2) On line 66, the authors should consider replacing “survey” with “intervention” or “program”

Answer: We replaced “survey” with “program.” (line 69)

3) On line 84, is it correct to interpret exclusion criteria #2 as “only a subset of students….”

Answer: Thank you for highlighting the ambiguity in our manuscript regarding the exclusion criteria. Your interpretation is indeed correct. We have revised the phrase.

A. 2) only a subset of the students in the target grade (e.g., only female students in a co-educational school) attended the programs; (line 91-93)

4) On line 85, it would help if the authors provided a short explanation as to why exclusion criteria #4 is important.

Answer: Thank you for seeking further clarification on exclusion criterion #4. As previously mentioned in our response to the major issue (4), this criterion is pivotal for accurately classifying participants into the 'cancer education' and 'control' groups.

5) The acronym OECD on line 91 needs to be spelled out as many readers may not know what that is.

Answer: We should not have used the abbreviation but spelled it out.

A. the Organisation for Economic Co-operation and Development (line 107-108)

6) For the survey (lines 100-101), were all questions Likert, were any open-ended questions used to better understand the impact of the program. For instance, “when did you first become aware of the importance of cancer screening”?

Answer: Thank you for your inquiry regarding the format of our survey questions. Our survey exclusively consisted of closed-ended questions. This approach was chosen to facilitate straightforward analysis and comparison of responses. Specifically, regarding the question of cancer screening intention, we provided details in the method section as mentioned in the major issue (3).

A. They provided their consents electronically and answered approximately 30 close-ended questions. (line 120-121)

A. Specifically, we added a novel question to gauge participants' attitudes towards future cancer screenings, utilizing a 6-point Likert scale for detailed insights. This question was formulated as follows:

Please select the attitude, most applicable to you, on undergoing cancer screening in the future.

(1, very low; 2, moderately low; 3, slightly low; 4, slightly high; 5, moderately high; 6, very high) (line 99-105)

7) On line 108, were these questions written with the term “recommended” before them which could potentially influence responses. Or, were they written as (a) never smoked and (b) have smoked.

Answer: Thank you for your question regarding the phrasing of our survey questions on healthy lifestyles. We designed these questions based on a previous Japanese study [15], ensuring that they were neutrally worded to avoid influencing the respondents' answers. It is important to note that the term 'recommended' was used only for categorizing responses in our analysis and not contained in the actual survey questions.

A. We set five items of a healthy lifestyle, which are nationally recommended specifically for cancer prevention, as binary variables of recommended (1) and non-recommended (0): smoking (recommended: never smoked), drinking alcohol (recommended: weekly alcohol consumption of < 150 g), exercising (recommended: ≥ 37.5 and ≥ 31.9 metabolic equivalent hours per day for men and women, respectively), eating salted meals (recommended: consumption of < 0.67 g of fish roe per day), and being overweight (recommended: body mass index [BMI] within the range of 21–27 for men and 19–25 for women); these thresholds were based on a previous Japanese study [11]. The term “recommended” was used only for categorizing responses in our analysis and not included in the actual survey questions. (line 126-134)

8) On lines 113-114, it may be helpful to provide additional information in the sentence on if the thresholds were set to reduce cancer risk or just for general healthy living.

Answer: Thank you for suggesting that we provide more context about the healthy lifestyle thresholds mentioned in our manuscript We should have clarified these healthy lifestyles were commended by the government to prevent cancer not to enhance general health. We have revised the relevant sentence in the methods section.

A. We set five items of a healthy lifestyle, which are nationally recommended specifically for cancer prevention, as binary variables of recommended (1) and non-recommended (0) (line 126-127)

9) On lines 119 and 121, it would be helpful to add in “logistic” to describe the regression

Answer: Thank you for bringing to our attention the terminology used to describe our statistical method. Upon review, we realized that we incorrectly referred to our regression analysis as 'logistic regression' when, in fact, it should have been described as 'linear regression'. We appreciate your keen observation, which has helped us identify and rectify this error. We have corrected the manuscript to accurately reflect the type of regression analysis used.

10) On line 120, it would be helpful to describe the “half-split” further or provide a reference for when/how it is used. That will be helpful for any reader attempting to replicate these methods.

Answer: We appreciate your attention to our use of the "half-split" approach in our study. To clarify, this technique involved splitting our dataset into two equal halves based on the healthy behavior scores, categorizing them from the lower scores upwards. In addition, as mentioned in our response to your major issue (3), we recognized that educational backgrounds, especially in healthcare or welfare, could have a significant impact on attitudes towards cancer screening. Consequently, we categorized this variable into two groups: 'healthcare or welfare education' and 'non-healthcare or welfare education.' We have revised the methodology section.

A. We used a half-split approach for categorizing healthy behavior scores, dividing the dataset into two equal halves from the lower values. (line 138-140)

A. Considering that educational background, particularly healthcare or welfare, could significantly influence participants' perspectives on health, we sought detailed responses in this area. (line 110-112)

11) On line 122, it would be beneficial to write, even parenthetically, the “variables that were not significant”. That will help the reader understand all parameters investigated.

Answer: Thank you for your comment. As mentioned in our response to your major issue (5), we have included Table 3 in the results section. This table details the outcomes of the t-test, including both significant and non-significant findings, thus offering a comprehensive view of all variables examined in our study.

12) For table 1, it would be helpful to describe what the column “time” is. The reader is assuming this is the time of the educational program, but it would be better to not leave that to interpretation.

Answer: Thank you for pointing out the potential ambiguity associated with the term "time" in Table 1. To eliminate any confusion and provide clear information, we have revised this term to "year".

13) Also, on table 1, there appears to be a typo “survery” instead of “survey”

Answer: We have also corrected the misspelled terms.

14) For table 2, would the authors be open to writing the age of the control as 21-24 instead of 21-22 & 23-24, or is there significance in writing the age as written?

Answer: Thank you for your suggestion regarding the age presentation in Table 2 of our manuscript. While our initial decision to present the ages as 21-22 & 23-24 was to align with the distribution of the control group compared to the cancer education group (22-23 years), we agree that simplifying this to a single range of 21-24 years is equally acceptable and may improve clarity for readers.

15) On lines 153-162, were multiple t-tests run, an ANOVA/MANOVA, or were the correlations part of the SPSS outcome from the logistic regression protocol?

Answer: Thank you for pointing out the ambiguity of our result. In the mentioned section, we primarily discussed the result of our multiple linear regression analysis. The results of this analysis are detailed in what is now Table 4 of our revised manuscript (previously referred to as Table 3 in the original version). The addition of Table 3 and the subsequent adjustment will make it easier for readers to understand the flow and outcomes of our statistical analysis.

16) Could the authors explain line 165. Is it a reference to the total number of participants, and if so, what is the correlation between?

Answer: Thank you for your inquiry regarding line 165 of our manuscript. We apologize for any confusion caused by our explanation in this section. To clarify, line 165 refers to a footnote associated with Table 4, where we discuss the results of our multiple linear regression analysis. In this context, 'R²' refers to the coefficient of determination, which is a statistical measure of how well the regression predictions match the actual data points. In our study, the adjusted R² value is 0.10, indicating that the model explains 10% of the variance in the outcome. While this suggests that the model provides some insight, it also suggests that the fit is not particularly strong.

We hope this explanation clarifies the content of line 165 and the nature of the correlation we report in our analysis.

17) On lines 173-174, the authors may want to comment on if the cancer education program could have influenced the health literacy score.

Answer: Thank you for your recommendation. Based on your suggestion, we have revised the discussion section to explore this relationship more explicitly.

A. Attending cancer education programs did not affect cancer screening intention but significantly improved health literacy scores, whereas healthcare or welfare educational background and health literacy scores significantly improved cancer screening intention. (line 213-215)

18) On lines 178-180, were any of the studies cited focused on behavioral change, as is the focus of this paper. If so, the authors could further discuss how their work fits into the literature.

Answer: Thank you for highlighting the relevance of behavioral change in the context of our study and its relation to existing literature. We have revised the discussion section.

A. Other studies have conducted assessments immediately after the cancer education class in the U.S., three months later in Korea, and six months later in the U.K., using original survey sheets, and confirmed improvements in knowledge and psychological aspects [8–10]. However, a study from Korea, which aligns more closely with the focus of our study, assessed healthy behavioral intentions to prevent cancer after an education program in elementary school students and reported no significant improvement after three months [9]. (line 217-222)

19) Lines 194-195, the paper could benefit from the authors commenting or addressing limitations on if the intention to undergo screening could be influenced by the participant’s health care provider/main physician for regular checkups.

Answer. Thank you for your insightful comments regarding the limitations of our study. It is important to consider the broader context of the Japanese healthcare system, where annual general health checkups are customary. This cultural norm may lead to an inherent expectation of cancer screenings as a routine practice, possibly influencing the high percentage of participants (approximately 90% in both the intervention and control groups) who indicated their intention to undergo cancer screening in the future. However, we recognize that the accuracy of these responses might be subject to bias, as the participants' current age and healthcare norms could diminish the perceived immediacy or personal relevance of such screenings.

To address these points, we have included the following sentences in the limitations section of our manuscript:

A. Considering the recommendations for routine cancer screenings in Japan, which typically commence at the age of 40, our study's participants, aged between 21 and 24, may not have perceived the screenings as practical. Approximately 90% of respondents in both the intervention and control groups expressed an intention to undergo cancer screening. However, it is noteworthy that these responses may be influenced by the prevailing healthcare attitudes in Japan, where annual health check-ups are standard [OECD Reviews of Public Health: Japan A HEALTHIER TOMORROW. OECD Publishing: Paris; 2019.]. Consequently, participants may have perceived cancer screenings as a routine rather than as a conscious health choice. (line 259-266)

20) For lines 196-197, did the 68% of students in that study complete the cancer education program?

Answer: Thank you for your question regarding the previous study referenced in our manuscript. To clarify, the earlier Japanese survey [20] focused on assessing students' willingness to undergo cancer screening and their sources of cancer information and understanding. This survey was not directly linked to participation in a cancer education program. To provide clearer context in our manuscript, we have revised the description as follows.

A. Second, in this study, the intention to undergo cancer screening in both groups was higher than expected. A previous Japanese survey, which only investigated cancer-screening intention, sources of cancer information, and level of cancer understanding among Japanese adolescents and was not linked to a cancer education program, reported that 68% of students indicated that they were willing to undergo cancer screening, in contrast to 91% in our study, although there were differences in the target age and questionnaires [20]. (line 239-244)

21) Lines 204-206, it could be helpful to briefly explain why one-on-one education provided additional benefit.

Answer. Thank you for the suggestion to include a more detailed explanation of educational styles in our discussion. We agree that clarifying the benefits of one-to-one educational approaches is important for understanding their effectiveness compared to group education. We have revised the relevant section as follows.

A. Third, in a past review, one-on-one educational approach significantly improved cancer screening rates with more robust evidence than group education because a personalized approach allows for the consideration of each participant's unique psychological and behavioral characteristics [22]. (line 250-253)

22) Lines 212-213, how many of the eligible participants did the school contact?

Answer. Thank you for inquiring about the extent of the school's outreach to eligible participants for our study. The cooperating school sent a total of 600 invitations. However, it's important to note that the address lists used for this outreach were based on information registered at the time of their graduation. This could mean that some of the addresses were outdated or incorrect, potentially leading to difficulties in reaching the intended recipients. In light of this, we have revised the limitations section of our manuscript to reflect these potential issues.

A. Furthermore, the survey participation rate of the target schools was approximately 20 percent. This low response rate may have been partly due to the school's reliance on graduation address lists, which may have had out-of-date or incorrect information. Therefore, some invitations may not have successfully reached the intended participants. Unfortunately, we were not able to determine the exact reasons for the low response rate, as we could not verify which alumni had received the invitations. In addition, respondents' medical interests may have been generally high. (line 266-272)

23) Lines 221-222, it would be helpful to address the change in curriculum briefly, key learning objectives that were different, etc.

Answer. Thank you for suggesting that we provide a more detailed comparison between the "doctor visit" program conducted by The Japan Cancer Society and the current national cancer education programs. In our response to your major issue (1), we noted that the "doctor visit" program did not follow a concrete educational format, allowing for flexibility in content delivery. In contrast, the current national cancer education programs are more structured, with clearly defined curriculum guidelines. These programs encompass nine key cancer-related topics: : 1) causes of cancer; 2) cancer processes; 3) the current cancer situation in Japan; 4) cancer prevention strategies; 5) cancer screenings; 6) treatment options; 7) palliative care; 8) quality of life for patients with cancer; and 9) understanding and empathizing with patients with cancer. Additionally, these programs are designed to be conducted annually in every school by schoolteachers and visiting lecturers, including medical professionals and cancer survivors. We have revised our discussion section to include this information for better understanding.

A. The "doctor visit" program did not follow a concrete educational format, allowing for flexibility in content delivery. In contrast, the current national cancer education programs are more structured, with clearly defined curriculum guidelines [24]. These programs encompass nine key cancer-related topics: 1) causes of cancer; 2) cancer processes; 3) the current cancer situation in Japan; 4) cancer prevention strategies; 5) cancer screenings; 6) treatment options; 7) palliative care; 8) quality of life for patients with cancer; and 9) understanding and empathizing with patients with cancer [24]. Additionally, these programs are designed to be conducted in every school by school teachers and visiting lecturers, including medical professionals and cancer survivors [24].

(line 280-289)

Other comments
1) Before finalizing, the paper would benefit from a thorough review for minor typos and to improve flow of the document.

Answer: Thank you for your comment. We modified our manuscript and ordered English-proof reading again.
