# Supplementary material for: Comparing health literacy and behavioral changes in adulthood: A pilot study on alumni of a single school cancer education program
Source: PLoS One. 2024 May 29;19(5):e0304381. doi: 10.1371/journal.pone.0304381 (PMC11135696; doi:10.1371/journal.pone.0304381)
Supplement: Attachment — Submitted filename: Response to Reviewers.docx [file pone.0304381.s002.docx]

Boyen Huang, DDS, MHA, PhD
Academic Editor
PLOS ONE

April 5^th^, 2024

Dear Boyen Huang, and Reveiwers

Subject: Submission of revised paper PONE-D-23-29716 entitled "Comparing Health Literacy and Behavioral Changes in Adulthood: A Pilot Study on Alumni of a Single School Cancer Education Program".

**(We modified the title, referred by the reviewer’s comments.)**

Thank you very much for reviewing our manuscript and offering valuable advice. We have addressed your comments and revised the manuscript accordingly. We modified our manuscript and ordered English-proof reading again.

We would be glad to respond to any further questions and comments that you may have.

Thank you very much for your consideration.

Sincerely,

Masanari Minamitani.

Response to Reviewer #1:

Thank you for your comments. Our answers to your points are as follows.

Major Issues:

1. Outcome applicability: I still have significant concerns about the overall question being asked with this and, in its current form, I would not feel comfortable applying the data from this study in a manner that would influence an educational curricular or assessment design nor would I utilize this to inform public health interventions with a population (either from an educational intervention standpoint or in policy/procedure development). I am not certain of what we are truly measuring as, with the further definition of the educational intervention, it sounds that the educational intervention was highly heterogeneous from site to site with multiple instructors each providing different forms of cancer-education/prevention information and without a standardized framework of learning objectives or curricular interventions. With the level of heterogeneity here, I fear that we are essentially measuring more about characteristics related to the limited student population at one individual school that participated more than the impacts of any remote and heterogeneous educational intervention on cancer education or prevention. I also fear that, with the current design of the manuscript focusing on the impact of the cancer education intervention, that, with the currently identified research question in mind, the study design is inherently flawed and unable to provide a usable result unless the research question is adjusted. Please identify if I have misinterpreted the details of the delivery and content of the educational intervention. Your description in the reviewer response is more detailed than what is identified in line 202, if there is indeed the level of heterogeneity in delivery that you describe in the reviewer response, I would recommend updating the description in the manuscript around line 202. This seems to go into more detail describing the heterogeneity around line 280.

Answer. Thank you for your comprehensive and insightful feedback. I acknowledge the concerns you raised regarding the applicability of our study's outcomes, especially in relation to the heterogeneity of the cancer education program delivered across different sites. Indeed, the cancer education program at the time was in a trial phase, which led to some variations in content delivery. However, as endorsed by the Japan Cancer Society, the core concept aimed to unify the message across junior high schools in Japan, emphasizing critical points such as the lifetime risk of cancer, the importance of a healthy lifestyle and regular cancer screenings, the comparatively low screening uptake rates in Japan versus Europe and the U.S., and the specifics of HPV vaccination and cervical cancer screening starting ages.

Based on this concept, it was presumed that all lecturers would address cancer screening in a relatively consistent manner, thereby justifying our focus on measuring future willingness to undergo cancer screening and the history of cervical cancer screening as indicators of the program's effectiveness.

We recognize, however, that the original title and study's scope may have suggested an overgeneralization. To reflect the study’s intent and scope more accurately, we have revised the title to "Comparing Health Literacy and Behavioral Changes in Adulthood: A Pilot Study on Alumni of a Single School Cancer Education Program."

A. This program did not have a rigidly-prescribed instructional content. However, as endorsed by the Japan Cancer Society, the core concept aimed to unify the message across junior high schools in Japan, emphasizing critical points such as the lifetime risk of cancer, the importance of a healthy lifestyle and regular cancer screenings, the comparatively low screening uptake rates in Japan versus Europe and the U.S., and the specifics of HPV vaccination and cervical cancer screening starting ages. This concept guided visiting lecturers, including oncologists, nurses, and cancer survivors, to share their clinical experiences and insights n various formats such as lectures, workshops, and group activities, tailoerd to effectively educate children with the target grades. The sessions typically lasted between one and three hours on a single day. (Line 75-84)

A. The session consisted of a lecture and a workshop, which used clear and simple language to ensure that the students understand the material. Under the concept of the “doctor visit”, which was described in the introduction, the radiation oncologist focused on cancer progression, prevention, screenings, and treatment options. (Line 207-211)

A. While adhering partially to the Japan Cancer Society's concept by covering topics like cancer screening and HPV vaccination, the program allowed for a degree of flexibility in how content was delivered. As a result of the achievements of this program, the current national cancer education programs have became more structured, with clearly defined curriculum guidelines [24]. (Line 285-290)

2. Patient population: Related to major issue 1, I have significant concerns about the applicability of the data with the study population being so limited and being 1. a single gender, and 2, in such limited numbers, the applicability and validity of the data comes into question. Unless the study population can be expanded both in number and in demographics, this will be a significant limitation. I'm wondering if there may be an opportunity to reconnect with the school that had staffing issues (school 13) or if additional connections or resources could be provided to institutions that responded, this would assist in establishing a more diverse respondent population and thus more theoretically valid and applicable results. Given the limitations if this is the only response feasible, I would recommend refocusing the research question in some way to make the results more applicable.

Answer: Thank you for highlighting the study population and the concerns regarding the applicability and validity of this study. We fully acknowledge the limitations of the study's scale, the limited and single-gender participant from one school, and the challenges of widening this population. Despite repeated attempts to recruit additional schools through the Japan Cancer Society, we have received refusals. We think increasing the participants will be practically impossible.

We have emphasized that this study should be seen as a first trial designed to prepare for future research. Following your suggestions, we have added sentences at the end of the limitations section to explicitly acknowledge the narrow demographic scope of the study and the consequent limitations in generalizing our findings.

We appreciate your understanding of the challenges we've faced and the steps we've taken to address the limitations within our control. We believe that transparent acknowledgement of these limitations, together with the potential of our study to pioneer the field, provides a balanced view of the contributions of our work and areas for future exploration.

A. The program content at that time was different from what it is today. Again, the participants in the study are extremely limited - single gender, from a single school, with fewer than 100 participants - which restricts the generalizability of the findings. It is also impractical to apply these findings directly to educational curricula. It is important to recognize this study as an initial, pilot effort to provide the basis for future research. While the current research focused on measuring screening uptake and motivation, a pre- and post-intervention survey found improvements in knowledge of cancer morbidity, preventability, and common causes, which was aligned with the objectives of the program [17]. Future studies should consider evaluating both knowledge and behavioral change. There is an increasing need to evaluate the effectiveness of cancer education and the relevance of its content within current formal cancer education programs. Despite its limitations, we believe that this study serves as a valuable pioneering effort in this area. (Line 295-307)

3. Immediate survey results vs longer term results: Details of results from a pre and post course survey identified in line 211 would be important to expand upon and may contribute to the study results. Was it clear that the educational intervention met targets at the time of the intervention based upon the initial pre and post survey results?

Answer: Thank you for pointing out the relationship between short- and long-term evaluation of the intervention. As previously mentioned, the core concept of “doctor visit” was to unify the message across junior high schools in Japan, emphasizing critical points such as the lifetime risk of cancer, the importance of a healthy lifestyle and regular cancer screenings, the comparatively low screening uptake rates in Japan versus Europe and the U.S., and the specifics of HPV vaccination and cervical cancer screening starting ages. The immediate survey showed the improvement of knowlegde about lifetime cancer morbidity, most common causes of cancer, and cancer preventability. This survey, which was not originally designed for academic research, focused only on these three items, limiting our ability to analyse the results comprehensively. Nevertheless, these results suggest that the program was partially achieving its educational objectives at the time of intervention. However, it's important to recognize that, particularly in prevention, behavioural change is more important than mere knowledge acquisition. The follow-up evaluation of adults in this study only assessed behavioural changes, leaving the impact on knowledge unclear. There may be a significant educational effect on cancer-related knowledge that could be explored more thoroughly in future studies. With this perspective, we have updated the discussion section.

A. It is important to recognize this study as an initial, pilot effort to provide the basis for future research. While the current research focused on measuring screening uptake and motivation, a pre- and post-intervention survey found improvements in knowledge of cancer morbidity, preventability, and common causes, which was aligned with the objectives of the program [17]. Future studies should consider evaluating both knowledge and behavioral change. There is an increasing need to evaluate the effectiveness of cancer education and the relevance of its content within current formal cancer education programs. Despite its limitations, we believe that this study serves as a valuable pioneering effort in this area. (Line 299-307)

Minor issues:

1. Japanese cultural influence of results: Please expand upon the item listed in line 231 and 232 that Japanese individuals are less likely to seek cancer screening than other populations. I worry about over generalization here. If this is the case, how could this help to adjust the research question here to make this study outcomes more meaningful and applicable?

Answer: Thank you for pointing out the unique aspect of Japanese culture. A key aim of the cancer education program was to promote behavioral change, in particularly to increase the uptake of adult cancer screening. This focus was driven by the significantly lower uptake of cancer screening in Japan compared to other countries, as reported in reference [19 (Jpn J Clin Oncol. 2021)]. We cited this study to investigate whether the cancer education program could positively influence Japan's current low screening rates and thus serve as a potential role model for future behavioral change initiatives. Unfortunately, the inability of our study to directly measure cervical cancer screening uptake - instead only measuring willingness to participate in screening - may have influenced the results of the study.

2. Learning objectives for educational intervention: Could you provide further detail as to learning objectives or goals for the educational sessions offered in the initial intervention?

Answer: Thank you for emphasizing the importance of detailing the learning objectives for the educational sessions in our initial intervention. As outlined in our previous responses, the core learning objectives of the intervention were designed to unify the message across junior high schools in Japan, emphasizing critical points such as the lifetime risk of cancer, the importance of a healthy lifestyle and regular cancer screenings, the comparatively low screening uptake rates in Japan versus Europe and the U.S., and the specifics of HPV vaccination and cervical cancer screening starting ages. We believe that specifying these objectives in our response and manuscript helps clarify the intended outcomes of the educational intervention.

Response to Reviewer #2:

Thank you very much. We are grateful for your valuable comments.
