# Supplementary material for: Comparing health literacy and behavioral changes in adulthood: A pilot study on alumni of a single school cancer education program
Source: PLoS One. 2024 May 29;19(5):e0304381. doi: 10.1371/journal.pone.0304381 (PMC11135696; doi:10.1371/journal.pone.0304381)
Supplement: Attachment — Submitted filename: Response to Reviewers.docx [file pone.0304381.s003.docx]

Boyen Huang, DDS, MHA, PhD
Academic Editor
PLOS ONE

May 10, 2024

Dear Boyen Huang, and Reveiwers

Subject: Submission of revised paper PONE-D-23-29716 entitled "Comparing Health Literacy and Behavioral Changes in Adulthood: A Pilot Study on Alumni of a Single School Cancer Education Program".

Thank you very much for reviewing our manuscript and offering valuable advice. We have addressed your comments and revised the manuscript accordingly. We modified our manuscript and short title.

We would be glad to respond to any further questions and comments that you may have.

Thank you very much for your consideration.

Sincerely,

Masanari Minamitani.

Response to the Editor

Thank you for your comments. Our answers to your points are as follows.

1. In addition to Reviewer 1's comments on the revisions in text (as below), I would strongly suggest the authors further address the limitations reported in the manuscript. For example, what can you and future researchers do differently in study design, data collection and/or data analysis to minimize the impact from those limitations on the validity of this study? What caution should readers take when they interprete your data and conclusion?

Answer. Thank you for pointing out vital limitations and future directions of our manuscript. We must clarify future prospects on cancer education programs evaluation. According to the CDC’s Health Education Curriculum Analysis Tool (HECAT), Health Education Curriculum and Standards require well-defined learning objectives and assessment strategies that focus on health-related knowledge, attitudes, and skills. Although systemic cancer education has already begun in Japan, continuous evaluation and curriculum modification are essential for enhancement. Given the challenges of retrospective assessment, as represented in this study, a prospective evaluation research would be more desirable. Future studies should consider longitudinal follow-up to assess the sustainability of cancer knowledge gains before and after the programs. In addition, investigating the impact of different types of visiting lecturers on knowledge acquisition could provide valuable insights. Understanding the long-term persistence of knowledge, which is related to behavior, will clarify the factors contributing to the current study’s negative results and enhance the implications drawn from this research. We would like to address these issues in our future research efforts. We added the following sentences into the last part of the discussion.

A. Although systemic cancer education has already begun in Japan, continuous evaluation and curriculum modification are essential for enhancement. Given the challenges of retrospective assessment, as represented in this study, a prospective evaluation research would be more desirable. Future studies should consider longitudinal follow-up to assess the sustainability of cancer knowledge gains before and after the programs. In addition, investigating the impact of different types of visiting lecturers on knowledge acquisition could provide valuable insights. Understanding the long-term persistence of knowledge, which is related to behavior, will clarify the factors contributing to the current study’s negative results and enhance the implications drawn from this research. There is an increasing need to evaluate the impacts of cancer education and the relevance of its content within current formal cancer education programs. Despite its limitations, we believe that this study serves as a valuable pioneering effort in this area.

Response to Reviewer #1:

We are thankful for your points. We revised our manuscript.

Reviewer #1: Overall, I believe the authors have adapted the overall focus of the manuscript and the direction of the text in a way that more accurately identifies the study's scope, focus, and limitations. The limitations are significant, and I would leave it up to the editorial board to identify whether these limitations are enough to exclude the study from publication. This is a negative study and, from an education-focused research perspective, it could be helpful to highlight the point that it is incredibly important to consider educational outcome evaluations as a part of designing an educational intervention (if there is a desire to observe outcomes). It is not entirely clear to me if this was a retrospective effort driven opportunistically by the authors or whether the authors did have a role in the initial intervention (I apologize if I missed this, either way, this should be identified in the manuscript).

Answer. Thank you for your comprehensive and insightful comments. This study was indeed initiated as part of movement to evaluate the long-term impacts of school cancer education programs that had been introduced by the Japan Cancer Society on a trial basis. Strictly speaking, it was not planned when the "doctor visits" were first introduced. One of the authors, a launch member of this program, had anticipated the potential for future evaluations. Still, a concrete research plan was not established because the primary goals of this association were enlightenment and education, without an emphasis on research. However, when formal cancer education programs began across the country around 2020, the importance of the evaluation became more apparent, leading us to conduct this study. As pointed out, this is a retrospective study, and the study's retrospective design and the somewhat arbitrary nature of its methods and interpretation are significant limitations. Nevertheless, this research addresses a critical points of public health education. Considering this, some modifications were made to the limitation section.

A. Fourth, this study was not planned when the "doctor visits" were first introduced. One of the authors, a launch member of this program, had anticipated the potential for future evaluations. Still, a concrete research plan was not established because the primary goals of the Japan Cancer Society were enlightenment and education, without an emphasis on research. However, when formal cancer education programs began across the country around 2020, the importance of the evaluation became more apparent, leading us to conduct this study. We admit that the study was retrospective and contained some arbitrary nature of its methods and interpretation. Nevertheless, this research addresses a critical point of public health education.

Revisions in text:

Line 43: would recommend changing "no significant effects" to highlight that no significant impact on measured outcomes.

Answer. Thank you for your suggestion. We have revised the wording to "no significant impact on the measured outcomes."

Line 56: this seems like too profound of a statement, this seems like it was a regional educational intervention, may walk back the degree of this statement's grandeur.

Answer. We are appreciated. We modified this part.

A. Before this initiative, formal cancer education was not widespread, and it is assumed that a few schools dealt with cancer independently.

Line 59: this seems too narrow of a statement about the purpose of cancer education programs. Would eliminate or soften language.

Answer. Thank you. We have eliminated this part.

Line 310: I would argue that "effectiveness" is not the true measurement of this, it sounds more consistent with understanding if there was a subject's behavior that would be consistent with recommendations of the educational intervention. This is a surrogate the for efficacy element.

Answer. We agree with your observation. We have acknowledged that evaluating "effectiveness" might be an oversimplification and have emphasized that we assessed whether the education program led to changes in behaviors. We reviewed and modified relevant phrases to better reflect this focus.

Line 311: would change to nine years prior rather than nine years ago.

Answer. Thank you for your comments. We modified the word.
