# Supplementary material for: Comparing health literacy and behavioral changes in adulthood: A pilot study on alumni of a single school cancer education program
Source: PLoS One. 2024 May 29;19(5):e0304381. doi: 10.1371/journal.pone.0304381 (PMC11135696; doi:10.1371/journal.pone.0304381)
Supplement: Peer review history

# Peer review: Decision Letter 0

**DOI:** 10.1371/journal.pone.0304381.r001

**Authors:** Boyen Huang

18 Dec 2023

PONE-D-23-29716Evaluating the long-term effectiveness of school cancer education programs on behavior change: a first trial studyPLOS ONE

Dear Dr. minamitani,

Thank you for submitting your manuscript to PLOS ONE. After careful consideration, we feel that it has merit but does not fully meet PLOS ONE’s publication criteria as it currently stands. Therefore, we invite you to submit a revised version of the manuscript that addresses the points raised during the review process.

Please submit your revised manuscript by Feb 01 2024 11:59PM. If you will need more time than this to complete your revisions, please reply to this message or contact the journal office at plosone@plos.org. When you're ready to submit your revision, log on to https://www.editorialmanager.com/pone/ and select the 'Submissions Needing Revision' folder to locate your manuscript file.

Please include the following items when submitting your revised manuscript:

- A rebuttal letter that responds to each point raised by the academic editor and reviewer(s). You should upload this letter as a separate file labeled 'Response to Reviewers'.
- A marked-up copy of your manuscript that highlights changes made to the original version. You should upload this as a separate file labeled 'Revised Manuscript with Track Changes'.
- An unmarked version of your revised paper without tracked changes. You should upload this as a separate file labeled 'Manuscript'.

If you would like to make changes to your financial disclosure, please include your updated statement in your cover letter. Guidelines for resubmitting your figure files are available below the reviewer comments at the end of this letter.

If applicable, we recommend that you deposit your laboratory protocols in protocols.io to enhance the reproducibility of your results. Protocols.io assigns your protocol its own identifier (DOI) so that it can be cited independently in the future. For instructions see: https://journals.plos.org/plosone/s/submission-guidelines#loc-laboratory-protocols. Additionally, PLOS ONE offers an option for publishing peer-reviewed Lab Protocol articles, which describe protocols hosted on protocols.io. Read more information on sharing protocols at https://plos.org/protocols?utm_medium=editorial-email&utm_source=authorletters&utm_campaign=protocols.

We look forward to receiving your revised manuscript.

Kind regards,

Boyen Huang, DDS, MHA, PhD

Academic Editor

PLOS ONE

Journal requirements:

When submitting your revision, we need you to address these additional requirements.

1. Please ensure that your manuscript meets PLOS ONE's style requirements, including those for file naming. The PLOS ONE style templates can be found at

https://journals.plos.org/plosone/s/file?id=wjVg/PLOSOne_formatting_sample_main_body.pdf and

https://journals.plos.org/plosone/s/file?id=ba62/PLOSOne_formatting_sample_title_authors_affiliations.pdf

2. Note from Emily Chenette, Editor in Chief of PLOS ONE, and Iain Hrynaszkiewicz, Director of Open Research Solutions at PLOS: Did you know that depositing data in a repository is associated with up to a 25% citation advantage (https://doi.org/10.1371/journal.pone.0230416)? If you’ve not already done so, consider depositing your raw data in a repository to ensure your work is read, appreciated and cited by the largest possible audience. You’ll also earn an Accessible Data icon on your published paper if you deposit your data in any participating repository (https://plos.org/open-science/open-data/#accessible-data).

3. We note that you have indicated that data from this study are available upon request. PLOS only allows data to be available upon request if there are legal or ethical restrictions on sharing data publicly. For more information on unacceptable data access restrictions, please see http://journals.plos.org/plosone/s/data-availability#loc-unacceptable-data-access-restrictions.

In your revised cover letter, please address the following prompts:

a) If there are ethical or legal restrictions on sharing a de-identified data set, please explain them in detail (e.g., data contain potentially sensitive information, data are owned by a third-party organization, etc.) and who has imposed them (e.g., an ethics committee). Please also provide contact information for a data access committee, ethics committee, or other institutional body to which data requests may be sent.

b) If there are no restrictions, please upload the minimal anonymized data set necessary to replicate your study findings as either Supporting Information files or to a stable, public repository and provide us with the relevant URLs, DOIs, or accession numbers. For a list of acceptable repositories, please see http://journals.plos.org/plosone/s/data-availability#loc-recommended-repositories.

We will update your Data Availability statement on your behalf to reflect the information you provide.

[Note: HTML markup is below. Please do not edit.]

Reviewers' comments:

Reviewer's Responses to Questions

**Comments to the Author**

1. Is the manuscript technically sound, and do the data support the conclusions?

The manuscript must describe a technically sound piece of scientific research with data that supports the conclusions. Experiments must have been conducted rigorously, with appropriate controls, replication, and sample sizes. The conclusions must be drawn appropriately based on the data presented. 

Reviewer #1: Partly

Reviewer #2: Partly

**********

2. Has the statistical analysis been performed appropriately and rigorously? 

Reviewer #1: Yes

Reviewer #2: Yes

**********

3. Have the authors made all data underlying the findings in their manuscript fully available?

The PLOS Data policy requires authors to make all data underlying the findings described in their manuscript fully available without restriction, with rare exception (please refer to the Data Availability Statement in the manuscript PDF file). The data should be provided as part of the manuscript or its supporting information, or deposited to a public repository. For example, in addition to summary statistics, the data points behind means, medians and variance measures should be available. If there are restrictions on publicly sharing data—e.g. participant privacy or use of data from a third party—those must be specified.

Reviewer #1: Yes

Reviewer #2: Yes

**********

4. Is the manuscript presented in an intelligible fashion and written in standard English?

PLOS ONE does not copyedit accepted manuscripts, so the language in submitted articles must be clear, correct, and unambiguous. Any typographical or grammatical errors should be corrected at revision, so please note any specific errors here.

Reviewer #1: Yes

Reviewer #2: Yes

**********

5. Review Comments to the Author

Please use the space provided to explain your answers to the questions above. You may also include additional comments for the author, including concerns about dual publication, research ethics, or publication ethics. (Please upload your review as an attachment if it exceeds 20,000 characters)

Reviewer #1: This is important to consider for overall impact of cancer, education, screening and health in general, however, I think there are some limitations to study that may limit the applicability of these results in the real world.

Considering that largely young males were included in the survey portion of the study, there may be significant impact on the results given that this group rarely has needs for screening in general, and I'm unaware of any immediate needs for cancer screening outside of Circle cancer, screening and women. This may influence the results, although it is unclear to me what it is more less likely to respond that they are willing to consider screening in the future.

Relatively high willingness to have any sort of cancer screening in the future, and concerned that this overall willingness to undergo cancer screening also results. it is unclear to me whether this is a Japanese cultural connection, or that overall willingness tend to cancer has risen from what be historical controls.

It is also unclear to me what the specific intervention was regarding cancer education. The level of detail is sparse, and, as an educationalist, it is unclear to me, whether the lack of impact is due to limitations in the teaching or evaluation methods at the time of teaching. While this is not specifically part of the study, this contextual background is important to understand what Impacts the actual education program itself potentially have on the outcomes of this survey.

Reviewer #2: Summary of research and overall impression

This is an interesting article to investigate if school-based cancer education programs supported behavioral change (defined as likelihood to participate in cancer screening) in those participating. While many school-based programs only look at the impact immediately after the program or at most a few months afterwards, the authors of this study sought to understand a long term effect by surveying participants 9+ years post-program. Of the thirteen schools contacted, only one school agreed to participate and contact their students; this resulted in the participants being all male (all male school) and in an urban setting. The authors established study and control groups using students from the school which agreed to participate. Of the six hundred who received invitations to participate, thirty-eight fitting the study group and fifty-five fitting the control group responded and completed the survey. The authors detailed the results of the study and performed statistical analysis to investigate which factors were significant.

The authors should be commended for seeking to understand the long-term impact of program such as these. Only through evaluation and continuous improvement do programs improve so that their outcomes show more impact and better connect with their learning objectives. However, the paper would be strengthened if the authors provided additional information needed for the reader to better understand the education program, the content delivered and how it connects with the behavioral change being investigated, and the delivery of the material (was it a one day, one module, year long program; was it delivered using age-appropriate language; was a learning objective of the program to create understanding of preventative cancer behaviors; etc. ). Knowing this would provide the reader a good foundation on which to understand the rest of the paper/study, as well as potential consider how to perform a similar study on their own programs. It would also be beneficial to acknowledge that data requirements for t-test and logistic regression were reviewed and met.

Major issues

1. The paper would be strengthened if the authors provided details on the cancer education programs.

a. What age level (JH-2 is mentioned in the table, but knowing the age level of the students would be beneficial.

b. Was the program a one day, one module, one year event?

c. The authors state the program was delivered by a radiation oncologist. Is it known if the physician communicated in an age-appropriate language to increase the understanding/learning of the students.

2. In the abstract (line 31), it is mentioned that the Japan Cancer Society has conducted these education programs since 2011; however in the introduction (lines 51-53), it is mentioned that the education programs became part of the curriculum in 2016. Before 2016 (which is when the study/control participants were in school), it would be helpful to know if the delivery of information was standardized or more invited guest lecture and variable. For instance, was focus on preventive measure compliance a learning objective/goal of the program; this is not mentioned in the objectives mentioned (lines 54-55).

3. It would be helpful if the authors were able to comment on if the questionnaire used was mapped to the curricular objectives of the program in which the students participated or if it was based only on general health and cancer related questions (lines 87-88). For instance, were the items included on lines 91-95 included in the curricular program for the students, or are they general health questions.

4. The authors describe the control group as students from one year before and one year after the study group. It would be helpful to explain this in more detail. As written, the reader understands that education programs began in 2011 and more formally in 2016; so, if the education program was ongoing at the time of the study participants, how are students one year afterwards a control group. One year before could be students who did not have the program, but if the program was given in 2013 (table 1), would one year before and one year after students both have been exposed to the education program.

5. It would be beneficial to acknowledge that data requirements for t-test and logistic regression were reviewed and met.

6. On lines 129-130, it would be helpful to further explain. Were all surveys completed by May 1, or was the letter an invitation to participate and survey sent later. This will help the reader better understand the timeline set in line 125.

7. It would be helpful if the authors provide more detail on what “medical and welfare” educational background is, as the reader is not familiar with these terms.

8. On table 2, what is “past medical history” with options of “zero” or “any”?

9. Lines 216-217, as written, the reader understands this to mean that the validity of the participants in the study/control groups could be compromised. Therefore, it would be extremely helpful to reword and further explain the meaning.

10. Lines 226-227, as written the sentence is very lofty as it refers to “cancer education programs” in general, and this study is limited to one school, one program (which may not be similar to current programs?). It would be helpful to reword and focus on the implications of this studying while also understanding the limitations.

Minor issues

1. The authors may want to revisit the phrasing of the sentence on lines 56-57. The incidence of cancer could be due to genetic factors or factors outside the influence of the education programs. However, the mortality rate could be influenced by preventive screening.

2. On line 66, the authors should consider replacing “survey” with “intervention” or “program”

3. On line 84, is it correct to interpret exclusion criteria #2 as “only a subset of students….”

4. On line 85, it would help if the authors provided a short explanation as to why exclusion criteria #4 is important.

5. The acronym OECD on line 91 needs to be spelled out as many readers may not know what that is.

6. For the survey (lines 100-101), were all questions Likert, were any open-ended questions used to better understand the impact of the program. For instance, “when did you first become aware of the importance of cancer screening”?

7. On line 108, were these questions written with the term “recommended” before them which could potentially influence responses. Or, were they written as (a) never smoked and (b) have smoked.

8. On lines 113-114, it may be helpful to provide additional information in the sentence on if the thresholds were set to reduce cancer risk or just for general healthy living.

9. On lines 119 and 121, it would be helpful to add in “logistic” to describe the regression

10. On line 120, it would be helpful to describe the “half-split” further or provide a reference for when/how it is used. That will be helpful for any reader attempting to replicate these methods.

11. On line 122, it would be beneficial to write, even parenthetically, the “variables that were not significant”. That will help the reader understand all parameters investigated.

12. For table 1, it would be helpful to describe what the column “time” is. The reader is assuming this is the time of the educational program, but it would be better to not leave that to interpretation.

13. Also, on table 1, there appears to be a typo “survery” instead of “survey”

14. For table 2, would the authors be open to writing the age of the control as 21-24 instead of 21-22 & 23-24, or is there significance in writing the age as written?

15. On lines 153-162, were multiple t-tests run, an ANOVA/MANOVA, or were the correlations part of the SPSS outcome from the logistic regression protocol?

16. Could the authors explain line 165. Is it a reference to the total number of participants, and if so, what is the correlation between?

17. On lines 173-174, the authors may want to comment on if the cancer education program could have influenced the health literacy score.

18. On lines 178-180, were any of the studies cited focused on behavioral change, as is the focus of this paper. If so, the authors could further discuss how their work fits into the literature.

19. Lines 194-195, the paper could benefit from the authors commenting or addressing limitations on if the intention to undergo screening could be influenced by the participant’s health care provider/main physician for regular checkups.

20. For lines 196-197, did the 68% of students in that study complete the cancer education program?

21. Lines 204-206, it could be helpful to briefly explain why one-on-one education provided additional benefit.

22. Lines 212-213, how many of the eligible participants did the school contact?

23. Lines 221-222, it would be helpful to address the change in curriculum briefly, key learning objectives that were different, etc.

Other comments

1. Before finalizing, the paper would benefit from a thorough review for minor typos and to improve flow of the document.

**********

6. PLOS authors have the option to publish the peer review history of their article (what does this mean?). If published, this will include your full peer review and any attached files.

If you choose “no”, your identity will remain anonymous but your review may still be made public.

**Do you want your identity to be public for this peer review?** For information about this choice, including consent withdrawal, please see our Privacy Policy.

Reviewer #1: **Yes: **Myles Nickolich

Reviewer #2: No

**********

[NOTE: If reviewer comments were submitted as an attachment file, they will be attached to this email and accessible via the submission site. Please log into your account, locate the manuscript record, and check for the action link "View Attachments". If this link does not appear, there are no attachment files.]

While revising your submission, please upload your figure files to the Preflight Analysis and Conversion Engine (PACE) digital diagnostic tool, https://pacev2.apexcovantage.com/. PACE helps ensure that figures meet PLOS requirements. To use PACE, you must first register as a user. Registration is free. Then, login and navigate to the UPLOAD tab, where you will find detailed instructions on how to use the tool. If you encounter any issues or have any questions when using PACE, please email PLOS at figures@plos.org. Please note that Supporting Information files do not need this step.

# Author comment: Author response to Decision Letter 0

**DOI:** 10.1371/journal.pone.0304381.r002

17 Jan 2024

Boyen Huang, DDS, MHA, PhD

Academic Editor

PLOS ONE

Jan 15th, 2024

Dear Boyen Huang, and Reveiwers

Subject: Submission of revised paper PONE-D-23-29716 entitled "Evaluating the long-term effectiveness of school cancer education programs on behavior change: a first trial study".

Thank you very much for reviewing our manuscript and offering valuable advice. We have addressed your comments and revised the manuscript accordingly. We modified our manuscript and ordered English-proof reading again.

We would be glad to respond to any further questions and comments that you may have. 

Thank you very much for your consideration.

Sincerely,

Masanari Minamitani.

 

Response to Reviewer #1:

Thank you for your comments. Our answers to your points are as follows.

1) This is important to consider for overall impact of cancer, education, screening and health in general, however, I think there are some limitations to study that may limit the applicability of these results in the real world.

Considering that largely young males were included in the survey portion of the study, there may be significant impact on the results given that this group rarely has needs for screening in general, and I'm unaware of any immediate needs for cancer screening outside of Circle cancer, screening and women. This may influence the results, although it is unclear to me what it is more less likely to respond that they are willing to consider screening in the future.

Answer. Thank you for your insightful comments regarding the limitations of our study, particularly concerning the demographic characteristics of our survey participants and the implications for the real-world applicability of our results.

In our study, we focused on a single private all-boys school in Tokyo for our survey due to challenges in securing cooperation from other schools, primarily by concerns over privacy. We acknowledge that this focus has potentially influenced the outcomes, given that the participant group primarily comprised young males aged 21 to 24 years. This age group is typically less engaged in cancer screening, as the recommendations for routine screenings such as gastric, lung, and colorectal cancer in Japan commence at the age of 40. Consequently, the immediate practicality and relevance of these screenings may not have been apparent to our participants.

Furthermore, it is important to consider the broader context of the Japanese healthcare system, where annual general health checkups are customary. This cultural norm may lead to an inherent expectation of cancer screenings as a routine practice, possibly influencing the high percentage of participants (approximately 90% in both the intervention and control groups) who indicated their intention to undergo cancer screening in the future. However, we recognize that the accuracy of these responses might be subject to bias, as the participants' current age and healthcare norms could diminish the perceived immediacy or personal relevance of such screenings.

To address these points, we have included the following sentences in the limitations section of our manuscript:

A. Considering the recommendations for routine cancer screenings in Japan, which typically commence at the age of 40, our study's participants, aged between 21 and 24, may not have perceived the screenings as practical. Approximately 90% of respondents in both the intervention and control groups expressed an intention to undergo cancer screening. However, it is noteworthy that these responses may be influenced by the prevailing healthcare attitudes in Japan, where annual health check-ups are standard [OECD Reviews of Public Health: Japan A HEALTHIER TOMORROW. OECD Publishing: Paris; 2019.]. Consequently, participants may have perceived cancer screenings as a routine rather than as a conscious health choice. (line 259-266)

2) Relatively high willingness to have any sort of cancer screening in the future, and concerned that this overall willingness to undergo cancer screening also results. it is unclear to me whether this is a Japanese cultural connection, or that overall willingness tend to cancer has risen from what be historical controls.

Answer: Thank you for highlighting the observed high willingness to undergo cancer screening among study participants. In our study, we noted an unexpectedly high inclination among participants in both the intervention and control groups to undergo future cancer screenings. As discussed in our manuscript, we believe this may be attributed to several factors. Firstly, the participants in our study exhibited a slightly higher level of health literacy and more frequently had educational backgrounds in healthcare or welfare compared to the general population. This could inherently influence their attitudes towards health screenings, including cancer screening. Additionally, as mentioned in our previous response (comment 1), the participants, being young males, might perceive cancer screening as a routine aspect of healthcare. To better reflect these considerations, we have revised the discussion section of our manuscript.

A. Second, in this study, the intention to undergo cancer screening in both groups was higher than expected. A previous Japanese survey, which only investigated cancer-screening intention, sources of cancer information, and level of cancer understanding among Japanese adolescents and was not linked to a cancer education program, reported that 68% of students indicated that they were willing to undergo cancer screening, in contrast to 91% in our study, although there were differences in the target age and questionnaires [20]. (line 239-244)

A. Considering the recommendations for routine cancer screenings in Japan, which typically commence at the age of 40, our study's participants, aged between 21 and 24, may not have perceived the screenings as practical. Approximately 90% of respondents in both the intervention and control groups expressed an intention to undergo cancer screening. However, it is noteworthy that these responses may be influenced by the prevailing healthcare attitudes in Japan, where annual health check-ups are standard [OECD Reviews of Public Health: Japan A HEALTHIER TOMORROW. OECD Publishing: Paris; 2019.]. Consequently, participants may have perceived cancer screenings as a routine rather than as a conscious health choice. (line 259-266)

3) It is also unclear to me what the specific intervention was regarding cancer education. The level of detail is sparse, and, as an educationalist, it is unclear to me, whether the lack of impact is due to limitations in the teaching or evaluation methods at the time of teaching. While this is not specifically part of the study, this contextual background is important to understand what Impacts the actual education program itself potentially have on the outcomes of this survey.

Answer: Thank you for pointing out the need for a more detailed description of the specific cancer education intervention implemented in our study. Upon reflection, we recognize the importance of providing a comprehensive context for the educational content and methods employed, as these factors are crucial in understanding the potential impact of the program on the study outcomes.

In response to your feedback, we have thoroughly reviewed the records of the school's cancer education program and have updated the relevant sections of our manuscript to include more specific details about the intervention.

A. This program did not have a rigidly-prescribed instructional content. Instead, visiting lecturers, including oncologists, nurses, and cancer survivors, shared their clinical experiences and insights to the target grades in a way they felt was most appropriate for educating children. The sessions were delivered in various formats, including lectures, workshops, and group activities and typically lasted between one and three hours on a single day. (line 75-80)

A. At this school, a cancer education session was conducted on October 22, 2013 [17]. The program was delivered by a radiation oncologist and a cancer survivor. They interacted with 173 second-year junior high school students (ages 13-14). The session consisted of a lecture and a workshop, which used clear and simple language to ensure that the students understand the material. The radiation oncologist focused on cancer progression, prevention, screenings, and treatment options. The survivor shared personal experiences with stomach and thyroid cancer, as well as her son's experience with leukemia. The workshop segment encouraged students to reflect on the emotional impact of losing loved ones to cancer and to consider preventive measures for themselves and others. A pre-and post-course survey revealed significant improvements in students' understanding of cancer-related issues: lifetime cancer morbidity (correct response rate, pre 31.0%, post 100%), most common causes of cancer (pre 60.1%, post 97.0%), and cancer preventability (pre 62.5%, post 93.3%) [17]. (line 202-212)

 

Response to Reviewer #2:

Thank you very much. Your comments are appreciated. We modified our manuscript.

Major issues:

1) The paper would be strengthened if the authors provided details on the cancer education programs.

a. What age level (JH-2 is mentioned in the table, but knowing the age level of the students would be beneficial.

b. Was the program a one day, one module, one year event?

c. The authors state the program was delivered by a radiation oncologist. Is it known if the physician communicated in an age-appropriate language to increase the understanding/learning of the students.

Answer: Thank you for pointing out the need for a more detailed description of the specific cancer education intervention implemented in our study. Upon reflection, we recognize the importance of providing a comprehensive context for the educational content and methods employed, as these factors are crucial in understanding the potential impact of the program on the study outcomes.

In response to your feedback, we have thoroughly reviewed the records of the school's cancer education program and have updated the relevant sections of our manuscript to include more specific details about the intervention.

A. This program did not have a rigidly-prescribed instructional content. Instead, visiting lecturers, including oncologists, nurses, and cancer survivors, shared their clinical experiences and insights to the target grades in a way they felt was most appropriate for educating children. The sessions were delivered in various formats, including lectures, workshops, and group activities and typically lasted between one and three hours on a single day. (line 75-80)

A. At this school, a cancer education session was conducted on October 22, 2013 [17]. The program was delivered by a radiation oncologist and a cancer survivor. They interacted with 173 second-year junior high school students (ages 13-14). The session consisted of a lecture and a workshop, which used clear and simple language to ensure that the students understand the material. The radiation oncologist focused on cancer progression, prevention, screenings, and treatment options. The survivor shared personal experiences with stomach and thyroid cancer, as well as her son's experience with leukemia. The workshop segment encouraged students to reflect on the emotional impact of losing loved ones to cancer and to consider preventive measures for themselves and others. A pre-and post-course survey revealed significant improvements in students' understanding of cancer-related issues: lifetime cancer morbidity (correct response rate, pre 31.0%, post 100%), most common causes of cancer (pre 60.1%, post 97.0%), and cancer preventability (pre 62.5%, post 93.3%) [17]. (line 202-212)

2) 2. In the abstract (line 31), it is mentioned that the Japan Cancer Society has conducted these education programs since 2011; however in the introduction (lines 51-53), it is mentioned that the education programs became part of the curriculum in 2016. Before 2016 (which is when the study/control participants were in school), it would be helpful to know if the delivery of information was standardized or more invited guest lecture and variable. For instance, was focus on preventive measure compliance a learning objective/goal of the program; this is not mentioned in the objectives mentioned (lines 54-55).

Answer: Thank you for bringing to our attention the discrepancy in the timeline of cancer education programs as mentioned in introduction. We recognize the need to clarify the distinction between the national cancer education programs and the specific initiatives undertaken by the Japan Cancer Society in our study. In our revised introduction, we have included a more detailed explanation to distinguish these two programs.

A. The Japanese cancer education program began as part of the national school health curriculum following the revision of the Cancer Control Act in 2016 and a partial change in the Third Basic Plan to Promote Cancer Control Programs in 2018 [1,2]. The national program was implemented in phases, starting with elementary schools in 2020, followed by junior high schools in 2021, and high schools in 2022. Before this initiative, formal cancer education was not part of the standard school education in Japan. (line 51-56)

A. This program did not have a rigidly-prescribed instructional content. Instead, visiting lecturers, including oncologists, nurses, and cancer survivors, shared their clinical experiences and insights to the target grades in a way they felt was most appropriate for educating children. The sessions were delivered in various formats, including lectures, workshops, and group activities and typically lasted between one and three hours on a single day. (line 75-80)

3) It would be helpful if the authors were able to comment on if the questionnaire used was mapped to the curricular objectives of the program in which the students participated or if it was based only on general health and cancer related questions (lines 87-88). For instance, were the items included on lines 91-95 included in the curricular program for the students, or are they general health questions.

Answer: Thank you for your inquiry regarding the alignment of our survey questionnaire with the curricular objectives of the cancer education program. Please note that the questionnaire was primarily designed to gather general health and cancer-related information, rather than directly linking to the specific curriculum of the program.

A. A questionnaire was developed for the survey by referring to previous studies. We incorporated questions about the willingness to undergo cancer screening and a history of cervical cancer screening (only for women), which is recommended for women aged 20 years and above [12]. Specifically, we added a novel question to gauge participants' attitudes towards future cancer screenings, utilizing a 6-point Likert scale for detailed insights. This question was formulated as follows:

Please select the attitude, most applicable to you, on undergoing cancer screening in the future.

(1, very low; 2, moderately low; 3, slightly low; 4, slightly high; 5, moderately high; 6, very high)

Other items were sex, age, residence (classified into predominantly urban, intermediate rural, and predominantly rural according to the Organisation for Economic Co-operation and Development’s regional typology), working status, academic background, medical history, health literacy, and five health-related behaviors (smoking, drinking, diet, physical activity, and body shape) [7,11,15,16]. Considering that educational background, particularly healthcare or welfare, could significantly influence participants' perspectives on health, we sought detailed responses in this area. We used the Japanese translation of the short version of the European Health Literacy Survey Questionnaire (HLS-Q12), a popular scale for assessing health literacy on a scale of 0-50 [7,15]. Health-related behaviors were cancer prevention lifestyle recommendations by the Ministry of Health, Labor, and Welfare of Japan [1,11]. (line 96-115)

4) The authors describe the control group as students from one year before and one year after th

Attachment Submitted filename: Response to Reviewers.docx

# Peer review: Decision Letter 1

**DOI:** 10.1371/journal.pone.0304381.r003

**Authors:** Boyen Huang

27 Mar 2024

PONE-D-23-29716R1Evaluating the long-term effectiveness of school cancer education programs on behavioral change: a first trial studyPLOS ONE

Dear Dr. minamitani,

Thank you for submitting your manuscript to PLOS ONE. After careful consideration, we feel that it has merit but does not fully meet PLOS ONE’s publication criteria as it currently stands. Therefore, we invite you to submit a revised version of the manuscript that addresses the points raised during the review process. Please address the comments and suggestions made by Reviewer 1, as shown below.

Please submit your revised manuscript by May 11 2024 11:59PM. If you will need more time than this to complete your revisions, please reply to this message or contact the journal office at plosone@plos.org. When you're ready to submit your revision, log on to https://www.editorialmanager.com/pone/ and select the 'Submissions Needing Revision' folder to locate your manuscript file.

Please include the following items when submitting your revised manuscript:

- A rebuttal letter that responds to each point raised by the academic editor and reviewer(s). You should upload this letter as a separate file labeled 'Response to Reviewers'.
- A marked-up copy of your manuscript that highlights changes made to the original version. You should upload this as a separate file labeled 'Revised Manuscript with Track Changes'.
- An unmarked version of your revised paper without tracked changes. You should upload this as a separate file labeled 'Manuscript'.

If you would like to make changes to your financial disclosure, please include your updated statement in your cover letter. Guidelines for resubmitting your figure files are available below the reviewer comments at the end of this letter.

If applicable, we recommend that you deposit your laboratory protocols in protocols.io to enhance the reproducibility of your results. Protocols.io assigns your protocol its own identifier (DOI) so that it can be cited independently in the future. For instructions see: https://journals.plos.org/plosone/s/submission-guidelines#loc-laboratory-protocols. Additionally, PLOS ONE offers an option for publishing peer-reviewed Lab Protocol articles, which describe protocols hosted on protocols.io. Read more information on sharing protocols at https://plos.org/protocols?utm_medium=editorial-email&utm_source=authorletters&utm_campaign=protocols.

We look forward to receiving your revised manuscript.

Kind regards,

Boyen Huang, DDS, MHA, PhD

Academic Editor

PLOS ONE

[Note: HTML markup is below. Please do not edit.]

Reviewers' comments:

Reviewer's Responses to Questions

**Comments to the Author**

1. If the authors have adequately addressed your comments raised in a previous round of review and you feel that this manuscript is now acceptable for publication, you may indicate that here to bypass the “Comments to the Author” section, enter your conflict of interest statement in the “Confidential to Editor” section, and submit your "Accept" recommendation.

Reviewer #1: (No Response)

Reviewer #2: All comments have been addressed

**********

2. Is the manuscript technically sound, and do the data support the conclusions?

The manuscript must describe a technically sound piece of scientific research with data that supports the conclusions. Experiments must have been conducted rigorously, with appropriate controls, replication, and sample sizes. The conclusions must be drawn appropriately based on the data presented. 

Reviewer #1: Partly

Reviewer #2: Yes

**********

3. Has the statistical analysis been performed appropriately and rigorously? 

Reviewer #1: Yes

Reviewer #2: Yes

**********

4. Have the authors made all data underlying the findings in their manuscript fully available?

The PLOS Data policy requires authors to make all data underlying the findings described in their manuscript fully available without restriction, with rare exception (please refer to the Data Availability Statement in the manuscript PDF file). The data should be provided as part of the manuscript or its supporting information, or deposited to a public repository. For example, in addition to summary statistics, the data points behind means, medians and variance measures should be available. If there are restrictions on publicly sharing data—e.g. participant privacy or use of data from a third party—those must be specified.

Reviewer #1: Yes

Reviewer #2: Yes

**********

5. Is the manuscript presented in an intelligible fashion and written in standard English?

PLOS ONE does not copyedit accepted manuscripts, so the language in submitted articles must be clear, correct, and unambiguous. Any typographical or grammatical errors should be corrected at revision, so please note any specific errors here.

Reviewer #1: Yes

Reviewer #2: Yes

**********

6. Review Comments to the Author

Please use the space provided to explain your answers to the questions above. You may also include additional comments for the author, including concerns about dual publication, research ethics, or publication ethics. (Please upload your review as an attachment if it exceeds 20,000 characters)

Reviewer #1: Thank you for your revisions and further consideration of the focus of the manuscript.

Major Issues:

1. Outcome applicability: I still have significant concerns about the overall question being asked with this and, in its current form, I would not feel comfortable applying the data from this study in a manner that would influence an educational curricular or assessment design nor would I utilize this to inform public health interventions with a population (either from an educational intervention standpoint or in policy/procedure development). I am not certain of what we are truly measuring as, with the further definition of the educational intervention, it sounds that the educational intervention was highly heterogeneous from site to site with multiple instructors each providing different forms of cancer-education/prevention information and without a standardized framework of learning objectives or curricular interventions. With the level of heterogeneity here, I fear that we are essentially measuring more about characteristics related to the limited student population at one individual school that participated more than the impacts of any remote and heterogeneous educational intervention on cancer education or prevention. I also fear that, with the current design of the manuscript focusing on the impact of the cancer education intervention, that, with the currently identified research question in mind, the study design is inherently flawed and unable to provide a usable result unless the research question is adjusted. Please identify if I have misinterpreted the details of the delivery and content of the educational intervention. Your description in the reviewer response is more detailed than what is identified in line 202, if there is indeed the level of heterogeneity in delivery that you describe in the reviewer response, I would recommend updating the description in the manuscript around line 202. This seems to go into more detail describing the heterogeneity around line 280.

2. Patient population: Related to major issue 1, I have significant concerns about the applicability of the data with the study population being so limited and being 1. a single gender, and 2, in such limited numbers, the applicability and validity of the data comes into question. Unless the study population can be expanded both in number and in demographics, this will be a significant limitation. I'm wondering if there may be an opportunity to reconnect with the school that had staffing issues (school 13) or if additional connections or resources could be provided to institutions that responded, this would assist in establishing a more diverse respondent population and thus more theoretically valid and applicable results. Given the limitations if this is the only response feasible, I would recommend refocusing the research question in some way to make the results more applicable.

3. Immediate survey results vs longer term results: Details of results from a pre and post course survey identified in line 211 would be important to expand upon and may contribute to the study results. Was it clear that the educational intervention met targets at the time of the intervention based upon the initial pre and post survey results?

Minor issues:

1. Japanese cultural influence of results: Please expand upon the item listed in line 231 and 232 that Japanese individuals are less likely to seek cancer screening than other populations. I worry about over generalization here. If this is the case, how could this help to adjust the research question here to make this study outcomes more meaningful and applicable?

2. Learning objectives for educational intervention: Could you provide further detail as to learning objectives or goals for the educational sessions offered in the initial intervention?

Reviewer #2: Thank you for the additions to the manuscript; they provide clarity for the reader. The additional information (and table) on the statistical methods/results is helpful in understanding factors considered. The reader also appreciated the track changes version as that helped identify the new components.

**********

7. PLOS authors have the option to publish the peer review history of their article (what does this mean?). If published, this will include your full peer review and any attached files.

If you choose “no”, your identity will remain anonymous but your review may still be made public.

**Do you want your identity to be public for this peer review?** For information about this choice, including consent withdrawal, please see our Privacy Policy.

Reviewer #1: No

Reviewer #2: No

**********

[NOTE: If reviewer comments were submitted as an attachment file, they will be attached to this email and accessible via the submission site. Please log into your account, locate the manuscript record, and check for the action link "View Attachments". If this link does not appear, there are no attachment files.]

While revising your submission, please upload your figure files to the Preflight Analysis and Conversion Engine (PACE) digital diagnostic tool, https://pacev2.apexcovantage.com/. PACE helps ensure that figures meet PLOS requirements. To use PACE, you must first register as a user. Registration is free. Then, login and navigate to the UPLOAD tab, where you will find detailed instructions on how to use the tool. If you encounter any issues or have any questions when using PACE, please email PLOS at figures@plos.org. Please note that Supporting Information files do not need this step.

# Author comment: Author response to Decision Letter 1

**DOI:** 10.1371/journal.pone.0304381.r004

5 Apr 2024

Boyen Huang, DDS, MHA, PhD

Academic Editor

PLOS ONE

April 5th, 2024

Dear Boyen Huang, and Reveiwers

Subject: Submission of revised paper PONE-D-23-29716 entitled "Comparing Health Literacy and Behavioral Changes in Adulthood: A Pilot Study on Alumni of a Single School Cancer Education Program".

(We modified the title, referred by the reviewer’s comments.)

Thank you very much for reviewing our manuscript and offering valuable advice. We have addressed your comments and revised the manuscript accordingly. We modified our manuscript and ordered English-proof reading again.

We would be glad to respond to any further questions and comments that you may have. 

Thank you very much for your consideration.

Sincerely,

Masanari Minamitani.

 

Response to Reviewer #1:

Thank you for your comments. Our answers to your points are as follows.

Major Issues:

1. Outcome applicability: I still have significant concerns about the overall question being asked with this and, in its current form, I would not feel comfortable applying the data from this study in a manner that would influence an educational curricular or assessment design nor would I utilize this to inform public health interventions with a population (either from an educational intervention standpoint or in policy/procedure development). I am not certain of what we are truly measuring as, with the further definition of the educational intervention, it sounds that the educational intervention was highly heterogeneous from site to site with multiple instructors each providing different forms of cancer-education/prevention information and without a standardized framework of learning objectives or curricular interventions. With the level of heterogeneity here, I fear that we are essentially measuring more about characteristics related to the limited student population at one individual school that participated more than the impacts of any remote and heterogeneous educational intervention on cancer education or prevention. I also fear that, with the current design of the manuscript focusing on the impact of the cancer education intervention, that, with the currently identified research question in mind, the study design is inherently flawed and unable to provide a usable result unless the research question is adjusted. Please identify if I have misinterpreted the details of the delivery and content of the educational intervention. Your description in the reviewer response is more detailed than what is identified in line 202, if there is indeed the level of heterogeneity in delivery that you describe in the reviewer response, I would recommend updating the description in the manuscript around line 202. This seems to go into more detail describing the heterogeneity around line 280.

Answer. Thank you for your comprehensive and insightful feedback. I acknowledge the concerns you raised regarding the applicability of our study's outcomes, especially in relation to the heterogeneity of the cancer education program delivered across different sites. Indeed, the cancer education program at the time was in a trial phase, which led to some variations in content delivery. However, as endorsed by the Japan Cancer Society, the core concept aimed to unify the message across junior high schools in Japan, emphasizing critical points such as the lifetime risk of cancer, the importance of a healthy lifestyle and regular cancer screenings, the comparatively low screening uptake rates in Japan versus Europe and the U.S., and the specifics of HPV vaccination and cervical cancer screening starting ages.

Based on this concept, it was presumed that all lecturers would address cancer screening in a relatively consistent manner, thereby justifying our focus on measuring future willingness to undergo cancer screening and the history of cervical cancer screening as indicators of the program's effectiveness. 

We recognize, however, that the original title and study's scope may have suggested an overgeneralization. To reflect the study’s intent and scope more accurately, we have revised the title to "Comparing Health Literacy and Behavioral Changes in Adulthood: A Pilot Study on Alumni of a Single School Cancer Education Program." 

A. This program did not have a rigidly-prescribed instructional content. However, as endorsed by the Japan Cancer Society, the core concept aimed to unify the message across junior high schools in Japan, emphasizing critical points such as the lifetime risk of cancer, the importance of a healthy lifestyle and regular cancer screenings, the comparatively low screening uptake rates in Japan versus Europe and the U.S., and the specifics of HPV vaccination and cervical cancer screening starting ages. This concept guided visiting lecturers, including oncologists, nurses, and cancer survivors, to share their clinical experiences and insights n various formats such as lectures, workshops, and group activities, tailoerd to effectively educate children with the target grades. The sessions typically lasted between one and three hours on a single day. (Line 75-84)

 A. The session consisted of a lecture and a workshop, which used clear and simple language to ensure that the students understand the material. Under the concept of the “doctor visit”, which was described in the introduction, the radiation oncologist focused on cancer progression, prevention, screenings, and treatment options. (Line 207-211)

 A. While adhering partially to the Japan Cancer Society's concept by covering topics like cancer screening and HPV vaccination, the program allowed for a degree of flexibility in how content was delivered. As a result of the achievements of this program, the current national cancer education programs have became more structured, with clearly defined curriculum guidelines [24]. (Line 285-290)

2. Patient population: Related to major issue 1, I have significant concerns about the applicability of the data with the study population being so limited and being 1. a single gender, and 2, in such limited numbers, the applicability and validity of the data comes into question. Unless the study population can be expanded both in number and in demographics, this will be a significant limitation. I'm wondering if there may be an opportunity to reconnect with the school that had staffing issues (school 13) or if additional connections or resources could be provided to institutions that responded, this would assist in establishing a more diverse respondent population and thus more theoretically valid and applicable results. Given the limitations if this is the only response feasible, I would recommend refocusing the research question in some way to make the results more applicable.

Answer: Thank you for highlighting the study population and the concerns regarding the applicability and validity of this study. We fully acknowledge the limitations of the study's scale, the limited and single-gender participant from one school, and the challenges of widening this population. Despite repeated attempts to recruit additional schools through the Japan Cancer Society, we have received refusals. We think increasing the participants will be practically impossible. 

We have emphasized that this study should be seen as a first trial designed to prepare for future research. Following your suggestions, we have added sentences at the end of the limitations section to explicitly acknowledge the narrow demographic scope of the study and the consequent limitations in generalizing our findings.

We appreciate your understanding of the challenges we've faced and the steps we've taken to address the limitations within our control. We believe that transparent acknowledgement of these limitations, together with the potential of our study to pioneer the field, provides a balanced view of the contributions of our work and areas for future exploration.

A. The program content at that time was different from what it is today. Again, the participants in the study are extremely limited - single gender, from a single school, with fewer than 100 participants - which restricts the generalizability of the findings. It is also impractical to apply these findings directly to educational curricula. It is important to recognize this study as an initial, pilot effort to provide the basis for future research. While the current research focused on measuring screening uptake and motivation, a pre- and post-intervention survey found improvements in knowledge of cancer morbidity, preventability, and common causes, which was aligned with the objectives of the program [17]. Future studies should consider evaluating both knowledge and behavioral change. There is an increasing need to evaluate the effectiveness of cancer education and the relevance of its content within current formal cancer education programs. Despite its limitations, we believe that this study serves as a valuable pioneering effort in this area. (Line 295-307)

3. Immediate survey results vs longer term results: Details of results from a pre and post course survey identified in line 211 would be important to expand upon and may contribute to the study results. Was it clear that the educational intervention met targets at the time of the intervention based upon the initial pre and post survey results?

Answer: Thank you for pointing out the relationship between short- and long-term evaluation of the intervention. As previously mentioned, the core concept of “doctor visit” was to unify the message across junior high schools in Japan, emphasizing critical points such as the lifetime risk of cancer, the importance of a healthy lifestyle and regular cancer screenings, the comparatively low screening uptake rates in Japan versus Europe and the U.S., and the specifics of HPV vaccination and cervical cancer screening starting ages. The immediate survey showed the improvement of knowlegde about lifetime cancer morbidity, most common causes of cancer, and cancer preventability. This survey, which was not originally designed for academic research, focused only on these three items, limiting our ability to analyse the results comprehensively. Nevertheless, these results suggest that the program was partially achieving its educational objectives at the time of intervention. However, it's important to recognize that, particularly in prevention, behavioural change is more important than mere knowledge acquisition. The follow-up evaluation of adults in this study only assessed behavioural changes, leaving the impact on knowledge unclear. There may be a significant educational effect on cancer-related knowledge that could be explored more thoroughly in future studies. With this perspective, we have updated the discussion section.

A. It is important to recognize this study as an initial, pilot effort to provide the basis for future research. While the current research focused on measuring screening uptake and motivation, a pre- and post-intervention survey found improvements in knowledge of cancer morbidity, preventability, and common causes, which was aligned with the objectives of the program [17]. Future studies should consider evaluating both knowledge and behavioral change. There is an increasing need to evaluate the effectiveness of cancer education and the relevance of its content within current formal cancer education programs. Despite its limitations, we believe that this study serves as a valuable pioneering effort in this area. (Line 299-307)

Minor issues:

1. Japanese cultural influence of results: Please expand upon the item listed in line 231 and 232 that Japanese individuals are less likely to seek cancer screening than other populations. I worry about over generalization here. If this is the case, how could this help to adjust the research question here to make this study outcomes more meaningful and applicable?

Answer: Thank you for pointing out the unique aspect of Japanese culture. A key aim of the cancer education program was to promote behavioral change, in particularly to increase the uptake of adult cancer screening. This focus was driven by the significantly lower uptake of cancer screening in Japan compared to other countries, as reported in reference [19 (Jpn J Clin Oncol. 2021)]. We cited this study to investigate whether the cancer education program could positively influence Japan's current low screening rates and thus serve as a potential role model for future behavioral change initiatives. Unfortunately, the inability of our study to directly measure cervical cancer screening uptake - instead only measuring willingness to participate in screening - may have influenced the results of the study.

2. Learning objectives for educational intervention: Could you provide further detail as to learning objectives or goals for the educational sessions offered in the initial intervention?

Answer: Thank you for emphasizing the importance of detailing the learning objectives for the educational sessions in our initial intervention. As outlined in our previous responses, the core learning objectives of the intervention were designed to unify the message across junior high schools in Japan, emphasizing critical points such as the lifetime risk of cancer, the importance of a healthy lifestyle and regular cancer screenings, the comparatively low screening uptake rates in Japan versus Europe and the U.S., and the specifics of HPV vaccination and cervical cancer screening starting ages. We believe that specifying these objectives in our response and manuscript helps clarify the intended outcomes of the educational intervention.

 

Response to Reviewer #2:

Thank you very much. We are grateful for your valuable comments.

Attachment Submitted filename: Response to Reviewers.docx

# Peer review: Decision Letter 2

**DOI:** 10.1371/journal.pone.0304381.r005

**Authors:** Boyen Huang

26 Apr 2024

PONE-D-23-29716R2Comparing Health Literacy and Behavioral Changes in Adulthood: A Pilot Study on Alumni of a Single School Cancer Education ProgramPLOS ONE

Dear Dr. minamitani,

Thank you for submitting your manuscript to PLOS ONE. After careful consideration, we feel that it has merit but does not fully meet PLOS ONE’s publication criteria as it currently stands. Therefore, we invite you to submit a revised version of the manuscript that addresses the points raised during the review process.

In addition to Reviewer 1's comments on the revisions in text (as below), I would strongly suggest the authors further address the limitations reported in the manuscript. For example, what can you and future researchers do differently in study design, data collection and/or data analysis to minimize the impact from those limitations on the validity of this study? What caution should readers take when they interprete your data and conclusion? 

Please submit your revised manuscript by Jun 10 2024 11:59PM. If you will need more time than this to complete your revisions, please reply to this message or contact the journal office at plosone@plos.org. When you're ready to submit your revision, log on to https://www.editorialmanager.com/pone/ and select the 'Submissions Needing Revision' folder to locate your manuscript file.

Please include the following items when submitting your revised manuscript:

- A rebuttal letter that responds to each point raised by the academic editor and reviewer(s). You should upload this letter as a separate file labeled 'Response to Reviewers'.
- A marked-up copy of your manuscript that highlights changes made to the original version. You should upload this as a separate file labeled 'Revised Manuscript with Track Changes'.
- An unmarked version of your revised paper without tracked changes. You should upload this as a separate file labeled 'Manuscript'.

If you would like to make changes to your financial disclosure, please include your updated statement in your cover letter. Guidelines for resubmitting your figure files are available below the reviewer comments at the end of this letter.

If applicable, we recommend that you deposit your laboratory protocols in protocols.io to enhance the reproducibility of your results. Protocols.io assigns your protocol its own identifier (DOI) so that it can be cited independently in the future. For instructions see: https://journals.plos.org/plosone/s/submission-guidelines#loc-laboratory-protocols. Additionally, PLOS ONE offers an option for publishing peer-reviewed Lab Protocol articles, which describe protocols hosted on protocols.io. Read more information on sharing protocols at https://plos.org/protocols?utm_medium=editorial-email&utm_source=authorletters&utm_campaign=protocols.

We look forward to receiving your revised manuscript.

Kind regards,

Boyen Huang, DDS, MHA, PhD

Academic Editor

PLOS ONE

Journal Requirements:

Please review your reference list to ensure that it is complete and correct. If you have cited papers that have been retracted, please include the rationale for doing so in the manuscript text, or remove these references and replace them with relevant current references. Any changes to the reference list should be mentioned in the rebuttal letter that accompanies your revised manuscript. If you need to cite a retracted article, indicate the article’s retracted status in the References list and also include a citation and full reference for the retraction notice.

[Note: HTML markup is below. Please do not edit.]

Reviewers' comments:

Reviewer's Responses to Questions

**Comments to the Author**

1. If the authors have adequately addressed your comments raised in a previous round of review and you feel that this manuscript is now acceptable for publication, you may indicate that here to bypass the “Comments to the Author” section, enter your conflict of interest statement in the “Confidential to Editor” section, and submit your "Accept" recommendation.

Reviewer #1: All comments have been addressed

**********

2. Is the manuscript technically sound, and do the data support the conclusions?

The manuscript must describe a technically sound piece of scientific research with data that supports the conclusions. Experiments must have been conducted rigorously, with appropriate controls, replication, and sample sizes. The conclusions must be drawn appropriately based on the data presented. 

Reviewer #1: Yes

**********

3. Has the statistical analysis been performed appropriately and rigorously? 

Reviewer #1: Yes

**********

4. Have the authors made all data underlying the findings in their manuscript fully available?

The PLOS Data policy requires authors to make all data underlying the findings described in their manuscript fully available without restriction, with rare exception (please refer to the Data Availability Statement in the manuscript PDF file). The data should be provided as part of the manuscript or its supporting information, or deposited to a public repository. For example, in addition to summary statistics, the data points behind means, medians and variance measures should be available. If there are restrictions on publicly sharing data—e.g. participant privacy or use of data from a third party—those must be specified.

Reviewer #1: Yes

**********

5. Is the manuscript presented in an intelligible fashion and written in standard English?

PLOS ONE does not copyedit accepted manuscripts, so the language in submitted articles must be clear, correct, and unambiguous. Any typographical or grammatical errors should be corrected at revision, so please note any specific errors here.

Reviewer #1: Yes

**********

6. Review Comments to the Author

Please use the space provided to explain your answers to the questions above. You may also include additional comments for the author, including concerns about dual publication, research ethics, or publication ethics. (Please upload your review as an attachment if it exceeds 20,000 characters)

Reviewer #1: Overall, I believe the authors have adapted the overall focus of the manuscript and the direction of the text in a way that more accurately identifies the study's scope, focus, and limitations. The limitations are significant, and I would leave it up to the editorial board to identify whether these limitations are enough to exclude the study from publication. This is a negative study and, from an education-focused research perspective, it could be helpful to highlight the point that it is incredibly important to consider educational outcome evaluations as a part of designing an educational intervention (if there is a desire to observe outcomes). It is not entirely clear to me if this was a retrospective effort driven opportunistically by the authors or whether the authors did have a role in the initial intervention (I apologize if I missed this, either way, this should be identified in the manuscript).

Revisions in text:

Line 43: would recommend changing "no significant effects" to highlight that no significant impact on measured outcomes.

Line 56: this seems like too profound of a statement, this seems like it was a regional educational intervention, may walk back the degree of this statement's grandeur.

Line 59: this seems too narrow of a statement about the purpose of cancer education programs. Would eliminate or soften language.

Line 310: I would argue that "effectiveness" is not the true measurement of this, it sounds more consistent with understanding if there was a subject's behavior that would be consistent with recommendations of the educational intervention. This is a surrogate the for efficacy element.

Line 311: would change to nine years prior rather than nine years ago.

**********

7. PLOS authors have the option to publish the peer review history of their article (what does this mean?). If published, this will include your full peer review and any attached files.

If you choose “no”, your identity will remain anonymous but your review may still be made public.

**Do you want your identity to be public for this peer review?** For information about this choice, including consent withdrawal, please see our Privacy Policy.

Reviewer #1: No

**********

[NOTE: If reviewer comments were submitted as an attachment file, they will be attached to this email and accessible via the submission site. Please log into your account, locate the manuscript record, and check for the action link "View Attachments". If this link does not appear, there are no attachment files.]

While revising your submission, please upload your figure files to the Preflight Analysis and Conversion Engine (PACE) digital diagnostic tool, https://pacev2.apexcovantage.com/. PACE helps ensure that figures meet PLOS requirements. To use PACE, you must first register as a user. Registration is free. Then, login and navigate to the UPLOAD tab, where you will find detailed instructions on how to use the tool. If you encounter any issues or have any questions when using PACE, please email PLOS at figures@plos.org. Please note that Supporting Information files do not need this step.

# Author comment: Author response to Decision Letter 2

**DOI:** 10.1371/journal.pone.0304381.r006

9 May 2024

Boyen Huang, DDS, MHA, PhD

Academic Editor

PLOS ONE

May 10, 2024

Dear Boyen Huang, and Reveiwers

Subject: Submission of revised paper PONE-D-23-29716 entitled "Comparing Health Literacy and Behavioral Changes in Adulthood: A Pilot Study on Alumni of a Single School Cancer Education Program".

Thank you very much for reviewing our manuscript and offering valuable advice. We have addressed your comments and revised the manuscript accordingly. We modified our manuscript and short title.

We would be glad to respond to any further questions and comments that you may have. 

Thank you very much for your consideration.

Sincerely,

Masanari Minamitani.

 

Response to the Editor

Thank you for your comments. Our answers to your points are as follows.

1. In addition to Reviewer 1's comments on the revisions in text (as below), I would strongly suggest the authors further address the limitations reported in the manuscript. For example, what can you and future researchers do differently in study design, data collection and/or data analysis to minimize the impact from those limitations on the validity of this study? What caution should readers take when they interprete your data and conclusion?

Answer. Thank you for pointing out vital limitations and future directions of our manuscript. We must clarify future prospects on cancer education programs evaluation. According to the CDC’s Health Education Curriculum Analysis Tool (HECAT), Health Education Curriculum and Standards require well-defined learning objectives and assessment strategies that focus on health-related knowledge, attitudes, and skills. Although systemic cancer education has already begun in Japan, continuous evaluation and curriculum modification are essential for enhancement. Given the challenges of retrospective assessment, as represented in this study, a prospective evaluation research would be more desirable. Future studies should consider longitudinal follow-up to assess the sustainability of cancer knowledge gains before and after the programs. In addition, investigating the impact of different types of visiting lecturers on knowledge acquisition could provide valuable insights. Understanding the long-term persistence of knowledge, which is related to behavior, will clarify the factors contributing to the current study’s negative results and enhance the implications drawn from this research. We would like to address these issues in our future research efforts. We added the following sentences into the last part of the discussion.

A. Although systemic cancer education has already begun in Japan, continuous evaluation and curriculum modification are essential for enhancement. Given the challenges of retrospective assessment, as represented in this study, a prospective evaluation research would be more desirable. Future studies should consider longitudinal follow-up to assess the sustainability of cancer knowledge gains before and after the programs. In addition, investigating the impact of different types of visiting lecturers on knowledge acquisition could provide valuable insights. Understanding the long-term persistence of knowledge, which is related to behavior, will clarify the factors contributing to the current study’s negative results and enhance the implications drawn from this research. There is an increasing need to evaluate the impacts of cancer education and the relevance of its content within current formal cancer education programs. Despite its limitations, we believe that this study serves as a valuable pioneering effort in this area.

Response to Reviewer #1:

We are thankful for your points. We revised our manuscript.

Reviewer #1: Overall, I believe the authors have adapted the overall focus of the manuscript and the direction of the text in a way that more accurately identifies the study's scope, focus, and limitations. The limitations are significant, and I would leave it up to the editorial board to identify whether these limitations are enough to exclude the study from publication. This is a negative study and, from an education-focused research perspective, it could be helpful to highlight the point that it is incredibly important to consider educational outcome evaluations as a part of designing an educational intervention (if there is a desire to observe outcomes). It is not entirely clear to me if this was a retrospective effort driven opportunistically by the authors or whether the authors did have a role in the initial intervention (I apologize if I missed this, either way, this should be identified in the manuscript).

Answer. Thank you for your comprehensive and insightful comments. This study was indeed initiated as part of movement to evaluate the long-term impacts of school cancer education programs that had been introduced by the Japan Cancer Society on a trial basis. Strictly speaking, it was not planned when the "doctor visits" were first introduced. One of the authors, a launch member of this program, had anticipated the potential for future evaluations. Still, a concrete research plan was not established because the primary goals of this association were enlightenment and education, without an emphasis on research. However, when formal cancer education programs began across the country around 2020, the importance of the evaluation became more apparent, leading us to conduct this study. As pointed out, this is a retrospective study, and the study's retrospective design and the somewhat arbitrary nature of its methods and interpretation are significant limitations. Nevertheless, this research addresses a critical points of public health education. Considering this, some modifications were made to the limitation section.

A. Fourth, this study was not planned when the "doctor visits" were first introduced. One of the authors, a launch member of this program, had anticipated the potential for future evaluations. Still, a concrete research plan was not established because the primary goals of the Japan Cancer Society were enlightenment and education, without an emphasis on research. However, when formal cancer education programs began across the country around 2020, the importance of the evaluation became more apparent, leading us to conduct this study. We admit that the study was retrospective and contained some arbitrary nature of its methods and interpretation. Nevertheless, this research addresses a critical point of public health education.

Revisions in text:

Line 43: would recommend changing "no significant effects" to highlight that no significant impact on measured outcomes. 

Answer. Thank you for your suggestion. We have revised the wording to "no significant impact on the measured outcomes."

Line 56: this seems like too profound of a statement, this seems like it was a regional educational intervention, may walk back the degree of this statement's grandeur.

 Answer. We are appreciated. We modified this part.

A. Before this initiative, formal cancer education was not widespread, and it is assumed that a few schools dealt with cancer independently.

Line 59: this seems too narrow of a statement about the purpose of cancer education programs. Would eliminate or soften language.　

 Answer. Thank you. We have eliminated this part.

Line 310: I would argue that "effectiveness" is not the true measurement of this, it sounds more consistent with understanding if there was a subject's behavior that would be consistent with recommendations of the educational intervention. This is a surrogate the for efficacy element.

Answer. We agree with your observation. We have acknowledged that evaluating "effectiveness" might be an oversimplification and have emphasized that we assessed whether the education program led to changes in behaviors. We reviewed and modified relevant phrases to better reflect this focus.

Line 311: would change to nine years prior rather than nine years ago.

Answer. Thank you for your comments. We modified the word.

Attachment Submitted filename: Response to Reviewers.docx

# Editor report: Decision Letter 3

**DOI:** 10.1371/journal.pone.0304381.r007

**Authors:** Boyen Huang

13 May 2024

Comparing Health Literacy and Behavioral Changes in Adulthood: A Pilot Study on Alumni of a Single School Cancer Education Program

PONE-D-23-29716R3

Dear Dr. minamitani,

We’re pleased to inform you that your manuscript has been judged scientifically suitable for publication and will be formally accepted for publication once it meets all outstanding technical requirements.

Within one week, you’ll receive an e-mail detailing the required amendments. When these have been addressed, you’ll receive a formal acceptance letter and your manuscript will be scheduled for publication.

An invoice will be generated when your article is formally accepted. Please note, if your institution has a publishing partnership with PLOS and your article meets the relevant criteria, all or part of your publication costs will be covered. Please make sure your user information is up-to-date by logging into Editorial Manager at Editorial Manager® and clicking the ‘Update My Information' link at the top of the page. If you have any questions relating to publication charges, please contact our Author Billing department directly at authorbilling@plos.org.

If your institution or institutions have a press office, please notify them about your upcoming paper to help maximize its impact. If they’ll be preparing press materials, please inform our press team as soon as possible -- no later than 48 hours after receiving the formal acceptance. Your manuscript will remain under strict press embargo until 2 pm Eastern Time on the date of publication. For more information, please contact onepress@plos.org.

Kind regards,

Boyen Huang, DDS, MHA, PhD

Academic Editor

PLOS ONE

Additional Editor Comments (optional):

Reviewers' comments:

# Editor report: Acceptance letter

**DOI:** 10.1371/journal.pone.0304381.r008

**Authors:** Boyen Huang

17 May 2024

PONE-D-23-29716R3 

PLOS ONE

Dear Dr. minamitani, 

I'm pleased to inform you that your manuscript has been deemed suitable for publication in PLOS ONE. Congratulations! Your manuscript is now being handed over to our production team.

At this stage, our production department will prepare your paper for publication. This includes ensuring the following:

* All references, tables, and figures are properly cited

* All relevant supporting information is included in the manuscript submission,

* There are no issues that prevent the paper from being properly typeset

If revisions are needed, the production department will contact you directly to resolve them. If no revisions are needed, you will receive an email when the publication date has been set. At this time, we do not offer pre-publication proofs to authors during production of the accepted work. Please keep in mind that we are working through a large volume of accepted articles, so please give us a few weeks to review your paper and let you know the next and final steps. 

Lastly, if your institution or institutions have a press office, please let them know about your upcoming paper now to help maximize its impact. If they'll be preparing press materials, please inform our press team within the next 48 hours. Your manuscript will remain under strict press embargo until 2 pm Eastern Time on the date of publication. For more information, please contact onepress@plos.org.

If we can help with anything else, please email us at customercare@plos.org.

Thank you for submitting your work to PLOS ONE and supporting open access. 

Kind regards, 

PLOS ONE Editorial Office Staff

on behalf of

Dr Boyen Huang 

Academic Editor

PLOS ONE
